# ‘Shared-Hook’ and ‘Changed-Hook’ Binding Activities of Herpesviral Core Nuclear Egress Complexes Identified by Random Mutagenesis

**DOI:** 10.3390/cells11244030

**Published:** 2022-12-13

**Authors:** Josephine Lösing, Sigrun Häge, Martin Schütz, Sabrina Wagner, Julia Wardin, Heinrich Sticht, Manfred Marschall

**Affiliations:** 1 Institute for Clinical and Molecular Virology, Friedrich-Alexander-Universität Erlangen-Nürnberg (FAU), 91054 Erlangen, Germany; 2 Division of Bioinformatics, Institute of Biochemistry, Friedrich-Alexander-Universität Erlangen-Nürnberg (FAU), 91054 Erlangen, Germany

**Keywords:** herpesviruses, human cytomegalovirus, nuclear egress complex (NEC), cytomegalovirus core NEC pUL50–pUL53, hook-into-groove-interaction, yeast two-hybrid screening, shared-hook binding activity, cross-viral NEC interactions, NEC as antiviral target, broad-spectrum antiviral strategy

## Abstract

Herpesviruses replicate their genomes and assemble their capsids in the host cell nucleus. To progress towards morphogenesis in the cytoplasm, herpesviruses evolved the strategy of nuclear egress as a highly regulated process of nucleo-cytoplasmic capsid transition. The process is conserved among α-, β- and γ-herpesviruses and involves the formation of a core and multicomponent nuclear egress complex (NEC). Core NEC is assembled by the interaction between the nucleoplasmic hook protein, i.e., pUL53 (human cytomegalovirus, HCMV), and the integral membrane-associated groove protein, i.e., pUL50. Our study aimed at the question of whether a panherpesviral NEC scaffold may enable hook-into-groove interaction across herpesviral subfamilies. For this purpose, NEC constructs were generated for members of all three subfamilies and analyzed for multi-ligand interaction using a yeast two-hybrid (Y2H) approach with randomized pUL53 mutagenesis libraries. The screening identified ten library clones displaying cross-viral shared hook-into-groove interaction. Interestingly, a slightly modified Y2H screening strategy provided thirteen further changed-hook pUL53 clones having lost parental pUL50 interaction but gained homolog interaction. In addition, we designed a sequence-predicted hybrid construct based on HCMV and Epstein-Barr virus (EBV) core NEC proteins and identified a cross-viral interaction phenotype. Confirmation was provided by applying protein–protein interaction analyses in human cells, such as coimmunoprecipitation settings, confocal nuclear rim colocalization assays, and HCMV ΔUL53 infection experiments with pUL53-complementing cells. Combined, the study provided the first examples of cross-viral NEC interaction patterns and revealed a higher yield of human cell-confirmed binding clones using a library exchange rate of 3.4 than 2.7. Thus, the study provides improved insights into herpesviral NEC protein binding specificities of core NEC formation. This novel information might be exploited to gain a potential target scaffold for the development of broadly acting NEC-directed inhibitory small molecules.

## 1. Introduction

Human cytomegalovirus (HCMV) is a worldwide distributed, major human pathogen, the pathogenicity of which has been underestimated for a long time. Infection of the immunocompetent host is predominantly limited to mild, typically mononucleosis-like symptoms [1], whereas HCMV infection can cause severe systemic and even life-threatening disease in immunosuppressed individuals, e.g., HIV-positives, tumor patients or transplant recipients [2]. The seroprevalence of HCMV ranges from approximately 40% to more than 95%, depending on the regional socio-economic status and hygiene standards of the population [3,4,5]. Most importantly, congenital HCMV infection (cCMV) represents the most frequent cause of pathogen-derived developmental defects of newborns and infants, such as sensorineural hearing loss, microcephaly or complex malformations of the cerebral convolutions [6,7,8]. The pathogenesis of HCMV infection is largely determined by the efficiency of viral replication and tissue-specific viral load, two factors that both depend on the manner of virus–host interaction. This property of a complex and multifaceted virus–host interaction is very similar for α-, β- and γ-herpesviruses, such as varicella zoster virus (VZV) [9], HCMV [4] and EBV [10].

**Figure 1:**
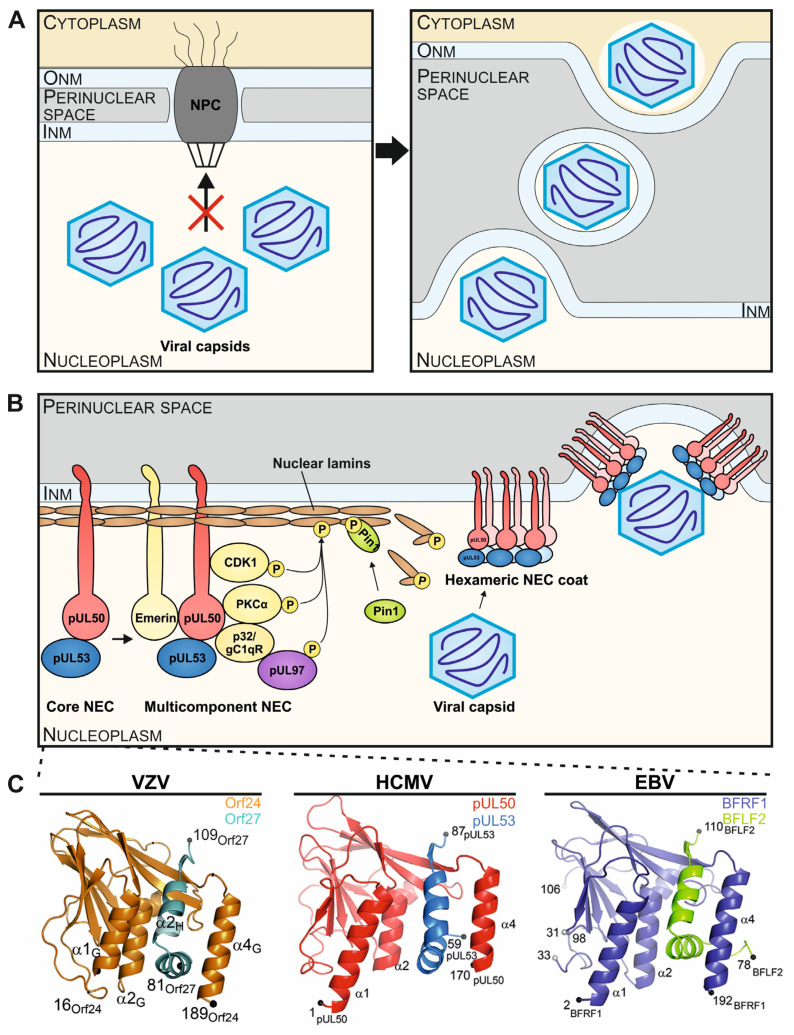
Illustration of the principle and multifunctional properties of the herpesviral nuclear egress complex (NEC) through the formation of core and multicomponent NEC arrangements. (**A**) Herpesviral capsids, assembled in the nucleus of infected cells, exceed the size exclusion limits of the nuclear pore complex (NPC), so that the fine-regulated process of nuclear egress evolved to enable the efficient nucleocytoplasmic transition of capsids through the nuclear envelope (INM and ONM, inner and outer nuclear membranes). (**B**) A schematic representation visualizes the stepwise formation of the HCMV-specific NEC, in its heterodimeric and hexameric core versions, and the multicomponent extension, including NEC-associated proteins. The core NEC, consisting of pUL50 and pUL53, recruits several cellular and viral proteins for the temporary reorganization of the nuclear lamina. A hexameric arrangement of the core NEC facilitates the docking and egress of viral capsids through the INM into the perinuclear space (refined illustrations referring to [20]). (**C**) Crystal structures of the VZV Orf24-Orf27, EBV BFRF1-BFLF2 and HCMV pUL50-pUL53 hook-into-groove formations in ribbon representations (refined illustrations referring to [14,18,19]).

Especially, the formation of multi-protein complexes consisting of viral and cellular proteins, such as multicomponent nuclear egress complexes (NECs), represent general determinants of herpesviral infection [11,12,13]. The fine-regulated nuclear egress is a process that enables the migration of the newly formed viral capsids from the nucleus to the cytoplasm. Viral nuclear egress leads to a massive reorganization of the nuclear envelope, which naturally represents a physical barrier for viral propagation due to its composition of the nuclear membranes, the nuclear lamina network, and the size limitation for nuclear transport of the nuclear pore complex (NPC; Figure 1A) [14,15]. Accordingly, an important property of the multifunctional NEC is to induce local disruption of the nuclear lamina and thus to facilitate the nucleocytoplasmic trafficking of viral capsids. As for all herpesviruses, the multicomponent NEC consists of both viral and cellular proteins that exert a number of enzymatic or regulatory properties (Figure 1B) [16]. The virus-encoded heterodimeric core complex of the NEC (core NEC) forms a central assembly scaffold and is defined by the interaction of the membrane-anchored protein homologs of HCMV pUL50, EBV BFRF1 or VZV Orf24, with their autologous, nucleoplasmic NEC partners pUL53, BFLF2 or Orf27, respectively. The high-affinity heterodimerization of core NEC proteins is based on conserved structural features, which are mostly characterized by a hook-into-groove interaction [17]. While both core NEC proteins adopt a globular fold with mixed secondary structure elements, the hallmark element of the interaction is an independent N-terminal hook-like extension of the nucleoplasmic core NEC protein. The hook segment consists of two consecutive α-helices followed by a short β-strand and is crucial for NEC core formation by contributing approximately 80% of the interaction surface. The interaction domain of the integral membrane protein is mainly composed of three α-helices, namely α1, α2 and α4 (plus the loop segment formed by α3 and β-sheet β9), and appears in a groove-like structure [14,17,18]. Interestingly, the crystal structures structures of core NECs are almost fully conserved, and the principle of hook-into-groove interaction is identical between all three herpesviral subfamilies (Figure 1C). In our previous studies, we were able to contribute to resolving the core NEC crystal structures of the α-, β- and γ-herpesviruses VZV, HCMV and EBV, respectively [14,18,19]. Of note, however, the degree of sequence conservation, in terms of amino acid identity, is very limited, thus merely ranging between 12.6% and 18.5% for the core NECs of VZV, HCMV and EBV [17,20].

On this basis, we underwent to study the cross-viral NEC interactions by various approaches [17,19,20]. In the present report, we used a Y2H on the basis of randomized mutagenesis libraries expressing HCMV pUL53 hook mutants and hybrid HCMV–EBV hook versions on a sequence prediction-assisted manner. We identified examples of shared-hook binding activity and properties of cross-viral hook-into-groove interaction. The findings enlarge our knowledge of herpesviral NEC protein binding specificity and may contribute to a strategy of developing broadly acting, possibly panherpesviral, NEC-directed inhibitory small molecules.

## 2. Materials and Methods

### 2.1. Cell Culture and Virus Infection

Human embryonic kidney epithelial 293T cells (HEK 293T, CRL-3216, ATCC, Manassas, VA, USA) and HeLa cells (ATCC) were cultivated in Dulbecco’s modified Eagle medium (DMEM, 11960044, Thermo Fisher Scientific, Waltham, MA, USA). Primary human foreskin fibroblasts (HFFs, C0045C, Thermo Fisher Scientific) were cultivated in a minimal essential medium (MEM, 21090022, Thermo Fisher Scientific). All cells were maintained at 37 °C, 5% CO_2_ and 80% humidity. Cell culture media were supplemented with 1× GlutaMAX™ (35050038, Thermo Fisher Scientific), 10 μg/mL gentamicin (22185.03, SERVA, Heidelberg, Germany) and 10% fetal bovine serum (FBS, FBS-12A, Capricorn Scientific, Ebsdorfergrund, Germany). For the cultivation of recombinant HFFs, tetracycline negative FBS (FBS-TET-12A, Capricorn Scientific) was used, and, additionally, 500 µg/mL of geneticin was added (G418, 10131035, Thermo Fisher Scientific).

For HCMV infection of recombinant HFFs, the cells were induced with doxycycline (Dox; D9891, Sigma-Aldrich, St. Louis, MO, USA) one day (d) prior to infection to induce protein expression. Recombinant HFF cells were inoculated with stocks of HCMV AD169/HB5-derived recombinant virus AD169-GFP ΔUL53 or its revertant HCMV Rev with equal genome amounts. After incubation for 90 min at 37 °C, the infectious supernatants were removed and replaced by fresh medium. Cells or supernatants were harvested at indicated time points for further analyses.

### 2.2. Plasmids and Transfection

Transient transfection was performed in 293T cells using polyethylenimine-DNA complexes (Sigma-Aldrich) as described previously [21]. HeLa cells were transfected by the use of FuGENE^®^ Transfection Reagent (Promega, Madison, WI, USA) according to the manufacturer’s instructions. 

The following plasmids were used for transfection: pDsRed1-N1 (RFP, Clontech, Kusatsu, Japan), pcDNA UL44-Flag (GPC Biotech AG, Martinsried, Germany), pcDNA-UL50-HA, pcDNA-UL53-Flag [22], pcDNA-HA-BFRF1 and pcDNA-Flag-BFLF2 (kindly provided by Chung-Pei Lee; National Taipei University of Nursing and Health Sciences, Taipeh, Taiwan).

Two randomized mutagenesis libraries coding for mutant versions of the pUL53 hook region (amino acids 55–87) that have an average of either 2.7 amino acid exchanges (library 1) or 3.4 exchanges (library 2) within the expression construct GAL4-AD::UL53(1–87)-Flag were purchased from GeneArt (Thermo Fisher Scientific, Regensburg, Germany). The manufacturer’s safety control resulted in 89% (library 1) or 92% (library 2) for library correctness. On average, each sequence contained in library 1 (Lib1): 3.8 nucleotide substitutions consisting of 1.5 transitions and 2.3 transversions and accordingly 2.7 amino acid substitutions; and contained in library 2 (Lib2): 4.8 nucleotide substitutions consisting of 1.8 transitions and 3.0 transversions and accordingly 3.4 amino acid substitutions. For the Y2H analyses, yeast codon-optimized (co) versions of HCMV pUL53 and pUL50 were also synthesized by GeneArt (Thermo Fisher Scientific).

Expression plasmids coding for untagged, N- or C-terminal HA-tagged or Flag-tagged VZV, herpes simplex virus type 1 (HSV-1), HCMV and EBV NEC homologs were generated by standard polymerase chain reaction (PCR) amplification of the respective template DNA. VZV (strain Oka), HSV-1 (strain 17^+^), HCMV (strain AD169) or EBV (strain B95-8) were used to generate the expression plasmids. Oligonucleotide primers (Table S1) used for PCR were purchased from Biomers (Ulm, Germany). After cleavage with the corresponding restriction enzymes, PCR products were inserted into the mammalian expression vector pcDNA3.1(+) (Life Technologies, Carlsbad, CA, USA), or the yeast expression vectors pGBT9 and pGAD424 (BD Biosciences Clontech, Mountain View, CA, USA).

### 2.3. Generation of Recombinant HFF Populations

#### 2.3.1. Generation of Lentiviral Expression Constructs

The lentiviral transfer plasmids for the generation of cells with inducible overexpression of pUL53 variants were constructed applying the Gateway cloning technology (Invitrogen, Waltham, MA, USA). Initially, the coding sequences for the open reading frame (ORF) UL53 and ORF UL53::sHook1 were amplified using pcDNA-UL53-Flag or pcDNA-UL53::sHook1-Flag as templates and the primers 5-UL53-attB1, 3-UL53-attB2 and 3-UL53-Flag-attB2. According to the manufacturer’s protocol, the attB-flanked UL53 sequences were transferred into the vector pDONR221 by a recombination reaction to create an entry clone and subsequently introduced into the mutated lentiviral expression plasmid pInducer20_CRSmut [23] by a second recombination step. The primers used for cloning reactions, 5-UL53-attB, 3-UL53-attB2 and 3-UL53-Flag-attB2, are depicted in Table S1.

#### 2.3.2. Lentiviral Transduction and Selection of Transduced Cells

For the generation of HFFs with an inducible overexpression of pUL53 variants, replication-deficient lentiviral particles were generated. To this end, HEK293 T cells were seeded in 10 cm dishes (5 × 10^6^ cells/dish) and transfected with the lentiviral expression plasmid pInducer20_CRSmut containing either the wild-type UL53, the Flag-tagged UL53 or the UL53::sHook1-Flag sequence together with the 3rd generation packaging plasmids pLP1, pLP2 and pLP-VSVg (Invitrogen) using the transfection reagent lipofectamine 2000 (11668019, Invitrogen). Supernatants containing lentiviral particles were harvested at 48 h after transfection, filtered (0.45 μm) to remove remaining cells and either directly used for lentiviral transduction or stored at −80 °C. HFFs of a low passage number were seeded in 6-well plates (8 × 10^4^ cells/well) and incubated for 24 h with lentiviral supernatants in the presence of 7.5 μg/mL polybrene (H9268, Sigma-Aldrich) to increase transduction efficiency. Transduced cells were selected at 48 h after transduction by the addition of 500 μg/mL geneticin to the cell culture medium. The expression of the recombinant proteins in the HFF cells was induced by the addition of 500 ng/mL of Dox, which was refreshed at least every third d.

### 2.4. Generation of HCMV ΔUL53 Using a Two-Step Markerless Red Recombination System

For the deletion of ORF UL53, a markerless bacterial artificial chromosome (BAC) mutagenesis [24] of the HCMV BAC HB5 AD169-GFP [25] was performed in *E. coli* strain GS1783 (kindly provided by Greg A. Smith, University of Chicago, IL, USA). The recombination was performed as described previously [23] by maintaining the overlapping regions of ORF UL53 with the ORFs UL52 and UL54 using the primers 5′ a-b-c-Kana and 3′ b-c-d-Kana (Table S1).

For the generation of infectious viral particles, HFFs of low passage were transfected with the respective recombinant BAC. One d prior to transfection, 3.0 × 10^5^ recombinant HFF UL53, HFF UL53-Flag and HFF UL53::sHook1-Flag were seeded into 6-well plates in sextuplicates. For transfection with the recombinant ΔUL53 BAC, FuGENE^®^ transfection reagent (Promega) was used. To this end, 1.5 μg BAC DNA and 0.25 μg of the plasmid pCB6-pp71 were mixed with 200 μL of MEM medium and 4 μL FuGENE^®^ (ratio 4:2 of FuGENE^®^:DNA). As a control construct, a UL53-positive revertant (HCMV Rev) was additionally generated by reinserting the ORF UL53 into the ΔUL53 BAC. The plasmid for the coexpression of HCMV tegument protein pp71 was used to overcome the repression of viral IE genes by ND10 [26,27]. The transfection mixture was incubated for 15 min at room temperature and added to the wells. After incubation for 4−6 h at 37 °C, the medium was replaced by fresh MEM. Approximately 7–10 days post-transfection (d p.t.), cells were transferred into 25 cm^2^ flasks and further incubated at 37 °C until plaques became microscopically visible, under weekly media changes. The supernatants of the successfully reconstituted virus were used for the infection of fresh HFFs and the preparation of virus stocks.

### 2.5. Yeast Two-Hybrid (Y2H) Analysis

For Y2H analyses, *S. cerevisiae* strain Y153 [28] was transformed by the lithium acetate method [29]. Hereby, 1.5 ml of YAPD medium [2% Bacto™ Yeast Extract (212750, Thermo Fisher Scientific), 4% Bacto™ Pepton (211677, Thermo Fisher Scientific) and 2% glucose] were inoculated with 15 μL of Y153 and grown overnight. All incubation steps were performed shaking at 30 °C. The Y153 culture was washed, and the cell pellet was resuspended in 500 μL lithium acetate solution (LiAc; 0.2 M) and incubated for 60 min. DNA of 0.25 up to 6 μg was prepared with 10 μl of carrier substance [reeved salmon sperm, denatured (95 °C, 10 min) and quenched on ice]. 140 μL of the yeast-LiAc suspension was added, and the samples were incubated for 30 min. Next, 350 μL of 50% PEG solution was added, and the mixture was incubated for a further 60 min. Then, a heat shock (42 °C, 15 min) was performed, and, after cooling to room temperature, the transformation reaction was washed two times with H_2_O. In a final step, the transformed yeast cells were resuspended in 100 μL H_2_O and plated on 20 mM 3-aminotriazole HWL^-^ plates. Plates were incubated at 30 °C for 6 to 9 days to allow selective growth of transformants. For large-scale Y2H screening, yeast strain Y153 was transformed by the lithium acetate method with bait plasmids as described above. These bait-positive Y153 cells were then used for large-scale transformation. Maintenance of the transformed bait plasmid was assured by selection for tryptophan prototrophy. For transformation, an amount of 0.25 up to 5 µg of the random mutagenesis libraries of truncated HCMV pUL53(1–87) was used per aliquot of 140 µl yeast cells. 

Protein interactions were analyzed using coexpressed GAL4 fusion proteins. Thereby, groove proteins were fused to the GAL4 binding domain (BD) and hook proteins, in particular the randomized mutagenesis libraries, were fused to the GAL4 activation domain (AD). In the case of a direct interaction, transcription of the reporter enzyme β-galactosidase was induced. The activity of β-galactosidase was determined by the filter lift assay [30]. To this end, the transformed colonies were transferred onto a nylon membrane Hybond™-N (GE Healthcare, Freiburg, Germany) and subsequently permeabilized by incubation for 1 min in liquid nitrogen. After direct thawing, the membrane was put on a layer of Whatman paper which was soaked in Xgal solution (60 mM Na_2_HPO_4_ × 7H_2_O, 42 mM NaH_2_PO_4_ × H_2_O, 10 mM KCl, 1 mM MgSO_4_ × 7H_2_O, 39 mM 2-mercaptoethanol, 0.8 mM X-β-gal) and incubated over night at 30 °C. 

**Figure 2:**
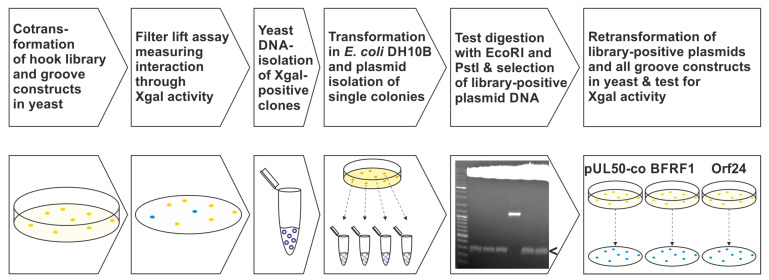
The methodological approach of the Y2H screening and further validation using a randomized pUL53 hook mutagenesis library and the groove constructs of VZV, HCMV and EBV. 1st and 2nd steps: cotransformation of the library with either pUL50, pUL50-co, BFRF1 or Orf24 in the yeast strain Y153 and measurement of Xgal activity. 3rd and fourth steps: library DNA isolation of interacting clones, transformation in bacteria and plasmid isolation of single colonies. 5th step: restriction enzyme digestion of plasmid DNA for selection of library DNA with an insert size of approximately 300 bp (i.e., confirming the hook construct), indicated by the arrow. 6th step: retransformation of confirmed library plasmids along with pUL50, pUL50-co, BFRF1 or Orf24, respectively, and testing for so-called shared-hook activity.

For the large-scale Y2H screening, in the filter lift assay, positive clones were further analyzed (Figure 2) by isolation of yeast DNA using the QIAprep Spin Miniprep Kit (QIAGEN, Hilden, Germany) according to the user-developed protocol (Michael Jones, Chugai Institute for Molecular Medicine, Ibaraki, Japan). The plasmids were rescued by the transformation of *E. coli* strain DH10B, isolation of single colony DNA and restriction digestion, followed by gel electrophoresis. Then, isolated library plasmids were retransformed into yeast to confirm interaction with the different groove proteins before sequences of the cDNA inserts were determined by automated sequence analysis (performed by Macrogen, Amsterdam, The Netherlands).

### 2.6. Antibodies

Antibodies used in this study: mAb-HA (Clone 7, H9658, Sigma-Aldrich); pAb-HA (Signalway Eurogentec, College Park, MD, USA); mAb-HA-HRP (12013819001, Roche, Basel, Switzerland); mAb-Flag (F1804, Sigma-Aldrich); mAb-Flag-HRP (A8592, Sigma-Aldrich); pAb-Histone H3 (PA5-17869, Invitrogen); mAb-Lamin A/C (sc-7292, Santa Cruz, Dallas, TX, USA); pAb-Aldolase (sc-12065, Santa Cruz); chicken Fc (chicken Fc fragment, 003-000-008, Jackson ImmunoResearch, Ely, UK); mAb-UL53.01 (kindly provided by Stipan Jonjic and Tihana Lenac Rovis, University of Rijeka, Rijeka, Croatia); anti-mouse Alexa 488 (A-11001, Thermo Fisher Scientific), anti-rabbit Alexa 555 (A-21428, Thermo Fisher Scientific).

### 2.7. Coimmunoprecipitation (CoIP), SDS-PAGE and Western Blot (Wb) Analyses

For the investigation of expression patterns, Dox-induced recombinant HFFs were harvested and lysed at the time points indicated. To analyze protein-protein interactions in a transient expression system, 293T cells were seeded into 10-cm dishes with a density of 5 × 10^6^ cells and transfected with expression plasmids. Three d p.t., CoIP was performed as described previously [31]. Immunoprecipitation control samples of approximately one-tenth of total volumes were taken prior to CoIP reactions. Antibody-coupled Dynabeads (25 µg/mL, 10002D, ThermoFisher Scientific) were used to obtain specific immunoprecipitates. Samples were subjected to the SDS–PAGE/Wb procedure as described previously [32].

### 2.8. Quantitative TaqMan Real-Time PCR (qPCR)

For viral replication kinetics, cells were infected with equal genome amounts and supernatants were collected at the indicated time points. The viral genome copy number was determined by IE1-specific (ORF UL123, exon 4) qPCR using a FAM/TAMRA-labeled probe, as described previously [33]. Each value given is a mean of quadruplicates ± standard deviations (SD).

### 2.9. Indirect Immunofluorescence (IF) Analysis and Confocal Laser-Scanning Microscopy

HEK293T or HeLa cells were seeded on coverslips for transfection. At two d p.t., cells were fixed and permeabilized following indirect immunofluorescence staining, as described previously [34]. Images were acquired with a TCS SP5 confocal laserscanning microscope (Leica Microsystems, Wetzlar, Germany) using the Leica LAS AF software (version 2.7.3.9723, Leica Microsystems CMS GmbH, Mannheim, Germany). The microscope was utilized with an HCX PL APO lambda blue 63x/NA 1.4 OIL objective, a 405 UV laser diode, an Argon laser, a 543 HeNe laser and a 633 HeNe laser, using monochrome filters with spectral ranges of 415–477, 496–540, 553–618 and 643–709, respectively. The device features a Leica photomultiplier tube (PMT) and a hybrid detector (HyD). Microscopic counting was performed for the quantitation of colocalization patterns of two different proteins. The criteria of counting were based on the distinctness of signals in positive cells, i.e., ‘complete’, those cells comprising a clearly defined or repeatedly found pattern of complete nuclear rim colocalization; ‘partial’, those comprising only partial colocalization patterns in individual cells; ‘no’, lack of colocalization. Unless otherwise indicated, two independent stainings were performed (biological duplicates), and 20–50 double-positive cells were used for countings performed twice from each staining area (technical duplicates, i.e., providing quadruplicate values for each).

### 2.10. HCMV Fluorescence-Based Replication Assay

For this assay, 8 × 10^4^ HFFs were cultivated in 24-well plates for the infection with HCMV ΔUL53 or HCMV Rev at a viral dose of 5 × 10^6^ genome copies. Cells were analyzed at indicated time points and subjected to automated GFP quantitation. For measurement of HCMV AD169-GFP ΔUL53-positive cells, signals were counted with the ImageXpress^®^ Pico device (Molecular Devices LLC, San Jose, CA, USA) using CellReporterXpress^®^ software (version 2.9.3.1183, Molecular Devices LLC) by the system-integrated cell count assay. The ImageXpress^®^ Pico device was utilized with a PL FLOUTAR 4x/NA 0.13 objective, bright field and digital confocal 2D light source, DAPI 350–390/419–482 and FITC 445–485/509–539 excitation and emission filters and the Sony CMOS detector.

## 3. Results

### 3.1. Expression Constructs and Experimental Approaches to Screen for Shared-Hook Binding Activity

#### 3.1.1. Rationale for the Generation of Sequence-Predicted Shared-Hook Constructs

**Figure 3:**
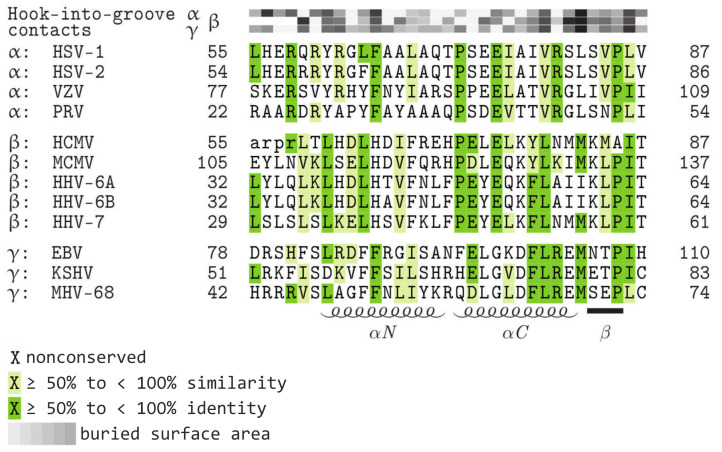
Sequence alignment of homologous hook proteins, highlighting conserved amino acids and amino acids representing contact interfaces. Increasing levels of sequence conservation are indicated by darker shades of green in the alignment. The buried surface area at hook-into-groove contact positions in α-, β-, and γ-herpesviruses is indicated by grey squares. Darker shades of grey indicate a larger buried surface. Lower-case letters mark those residues that were not resolved in the crystal structure (refined illustration referring to [17]). Note, that HCMV pUL53 is the only hook protein with an alanine at position 85, whereas the amino acid proline is conserved at this residue for the other hook proteins. The elements of the secondary structure are depicted schematically below the alignment. HSV-1, herpes simplex virus type 1; HSV-2, herpes simplex virus type 2; VZV, varicella zoster virus; PRV, pseudorabies virus; HCMV, human cytomegalovirus; MCMV, murine cytomegalovirus; HHV-6A, human herpesvirus 6A; HHV-6B, human herpesvirus 6B; HHV-7, human herpesvirus 7; EBV, Epstein-Barr virus; KSHV, Kaposi’s sarcoma-associated herpesvirus; MHV-68, murine herpesvirus 68.

Based on the conservation of shared structural features, particularly the hook-into-groove interaction interface, of core NEC proteins, we considered the possibility to generate hook constructs, on the basis of sequence-structure predictions, that may possess the ability to bind to various herpesviral groove proteins. To this end, we carefully inspected the underlying hook protein primary sequences of HCMV pUL53, EBV BFLF2 and VZV Orf27 and suggested hybrid-hook constructs with a putative shared-hook binding potential (Figure 3). Specifically, those contact amino acids that determine the hook-into-groove interaction were extracted from either of the three sequences for defining common consensus stretches, also according to their buried surface areas (Figure 3, upper lines, α-, β-, γ-herpesviral contacts). In addition, it was further attempted to include amino acids with a high identity grade across all herpesviral subfamilies (Figure 3, dark green). Consequently, construct UL53::sHook1, contained a hybrid-hook sequence adjusted between HCMV pUL53 (amino acids 55–87) and EBV BFLF2 (78–110), whereas UL53::sHook2 between HCMV pUL53 and VZV Orf27 (77–109). Thus, the final sequence-predicted hybrid constructs were primarily based on HCMV pUL53, including the respective replacements in their N-terminus, while the entire globular domain of the C-terminal part remained un-modified. Moreover, the constructs were C-terminally Flag-tagged to facilitate detection. Both sequences were cloned into the mammalian expression vector pcDNA3.1 (for cell culture-based interaction analysis) and the yeast vector pGAD424 (for Y2H analysis).

#### 3.1.2. Rationale for the Generation of Library-Based Shared-Hook Constructs

**Figure 4:**
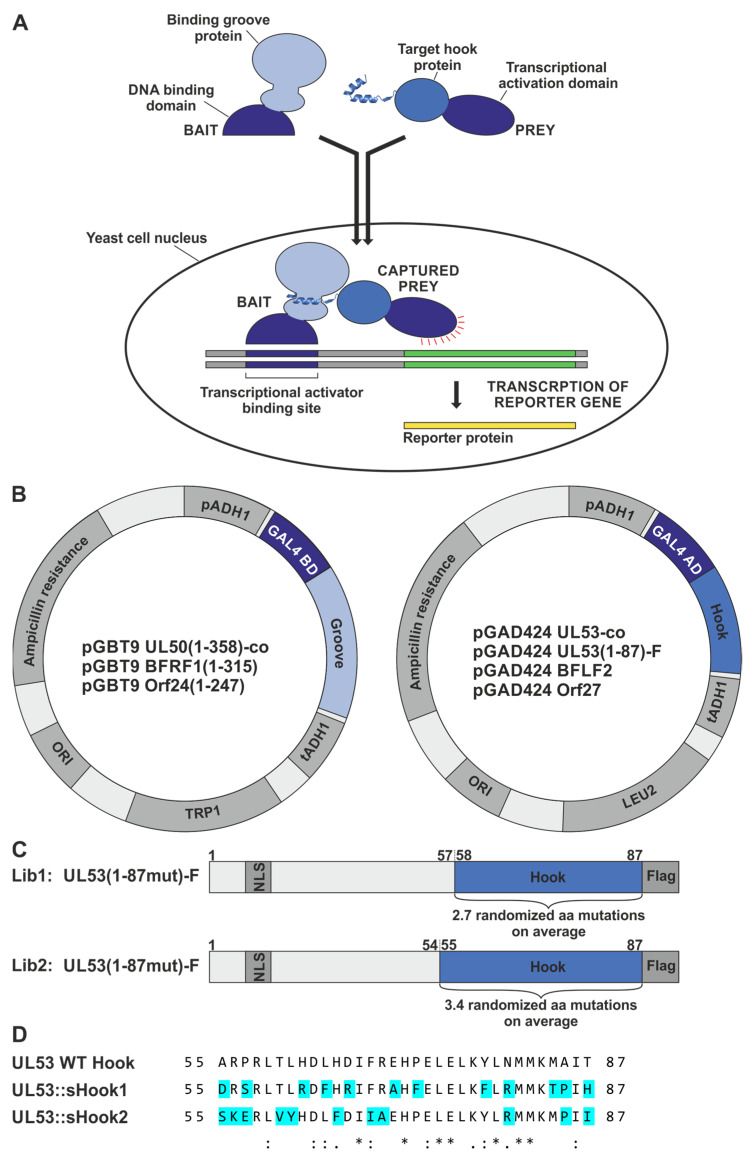
Schematic overview of the Y2H principle, yeast expression constructs and design of the hook mutagenesis libraries of truncated pUL53. (**A**) The Y2H system uses reporter genes to detect the interaction of proteins inside the yeast cell nucleus. The hook-into-groove interaction of a prey hook protein fused to the GAL4 activation domain (AD), with a bait groove protein fused to the GAL4 binding domain (BD), brings together the two-domain transcriptional activator, which then switches on the expression of reporter genes (illustration based on [35]). (**B**) Vector map of yeast expression constructs coding for fusion proteins of the GAL4 AD with hook proteins, here ORF UL53-co, ORF UL53(1–87)-Flag, ORF BFLF2 and Orf27, and the GAL4 BD fused to groove proteins lacking their transmembrane domain (TMD), here ORF UL50(1–358)-co, ORF BFRF1(1–315) and Orf24(1–247). The plasmids allow the selection of plasmid-positive clones for leucine (LEU2) or tryptophan (TRP1) auxotrophies in yeast, or ampicillin resistance in bacteria, respectively. Ori, origin of replication; pADH1/tADH1, transcriptional promoter/terminator region of the alcohol dehydrogenase gene 1. (**C**) Design of the hook mutagenesis libraries, Lib1 (amino acid mutation rate of 2.7) and Lib2 (rate of 3.4), expressing truncated pUL53-fusion versions with nuclear localization signal (NLS) and Flag-tag indicated in dark-grey. The hook region of pUL53 amino acids 58–87 for Lib1 and 55–87 for Lib2, containing 2.7 or 3.4 randomized amino acid mutations on average, is depicted in blue. (**D**) In addition to the randomized mutants of the libraries, two HCMV-EBV/VZV hybrid constructs were generated on the basis of primary sequence-predicted optimization. A comparative analysis of sequences was used to design these two hybrid constructs of a full-length-expressed pUL53 contained in the yeast vector pGAD424, in which binding-relevant amino acids of the construct sHook1 were preferentially adapted to a more EBV BFLF2-like fashion, while those of sHook2 were adapted to a more VZV Orf27-like fashion (for details, see Section 3.1.1). *, indicates positions of fully conserved residues; :, indicates conservation between groups of strongly similar properties (i.e., scoring > 0.5 in the Gonnet PAM 250 matrix); ., indicates conservation between groups of weakly similar properties (i.e., scoring ≤ 0.5 and > 0 in the Gonnet PAM 250 matrix).

Y2H screening was performed using two randomized mutagenesis libraries expressing GAL4 AD::hook fusion constructs of HCMV pUL53 (Figure 4). This version of the Y2H system utilized two reporter/selection systems of the yeast strain *S. cerevisiae* Y153 (Figure 4A). On the one hand, the HIS3 reporter enabled interaction-selected growth, and on the other hand, the lacZ reporter was used to measure interaction-dependent ß-galactosidase expression via the Xgal colour-staining signal (blue colonies). The readout of the screening was directed to hook-into-groove interaction as indicated by the positive reaction of the respective GAL4 BD::groove fusion constructs, either derived from HCMV pUL53 or EBV BFRF1 (Figure 4B). As additional positive controls, yeast codon-optimized pUL50 and pUL53 expression constructs were also generated, in order to overcome some limitations in achieving sufficiently high expression levels required for safe signal detection in the Y2H system (Figure 4B, pGBT9 UL50(1–358)-co and pGAD424 UL53-co).

Various rounds of screening were conducted using two randomized mutagenesis libraries expressing amino acids 1–87 (Figure 4C), i.e., representing the pUL53 hook region, in a substitution average of 2.7 (Lib1) or 3.4 (Lib2). This approach of random screening (Figure 4C), was compared with the analysis of abovementioned ‘designed’ sequence-predicted constructs (Figure 4D).

**Figure 5:**
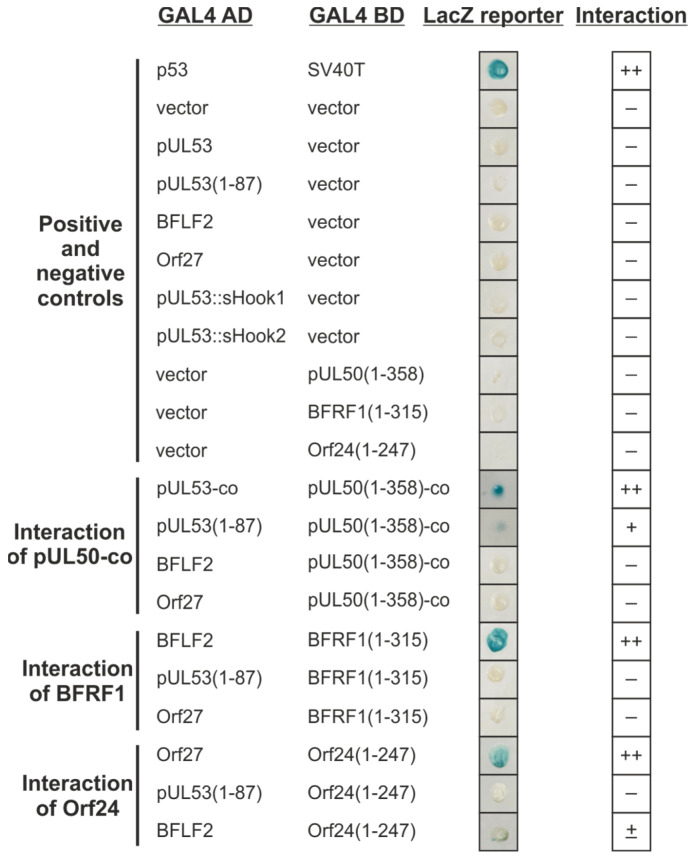
Y2H analysis of interactions between the hook and groove proteins of HCMV, EBV and VZV. The indicated hook proteins fused to the GAL4 AD, were cotransformed in various combinations with groove proteins fused to the GAL4 BD, into yeast strain Y153. The ß-galactosidase expression was detected with the filter lift assay. The known interaction of cellular tumor suppressor p53 with the simian virus 40 (SV40)-derived oncoprotein SV40T served as a positive control. The cotransformation of the empty vectors pGAD424 and pGBT9 with the constructs were used as a negative control. Interaction analysis of codon-optimized pUL50-co, BFRF1 and Orf24 with codon-optimized pUL53-co, pUL53(1–87), BFLF2 and Orf27, respectively. ++, indicates ≥ 50%; +, indicates < 50% Xgal-positive yeast colonies in the filter lift assay measuring interaction; –, indicates not interacting protein pairs.

As a first step, the positive and negative controls were assessed within this system, including empty vector control settings to rule out false positive reactions of novel clones with putative unintentional auto-activation (Figure 5). This control panel (using p53–SV40T as the positive reference, and vector–vector as the negative reference; Figure 5, upper part) clearly indicated that the produced constructs were functional, in that positive reactions were obtained for all autologous NEC pairs [pUL53-co and pUL50(1–358)-co; BFLF2 and BFRF1(1–315); Orf27 and Orf24(1–247); Figure 5, lower part].

### 3.2. Identification of Screening Hits in the Yeast Two-Hybrid System

In the next step, primary rounds of screening were performed by the use of the pUL53 mutagenesis library Lib1, as analyzed for interaction with either of the three partners, i.e., autologous pUL50 [performed in parallel with both wild-type (wt) and codon-optimized (co) pUL50], as well as nonautologous BFRF1 or Orf24 (Table 1). The starting quantity of yeast cells was comparable, in a range between 2.8–3.6 cells/0.5 µg of transformed library DNA. Interacting pUL53 library clones were scored in all cases, with numbers of initial positives between 3 and 28 clones. From most of these yeast clones, plasmid DNA could be successfully isolated and used for recovery in *E. coli*. All recovered (and reconfirmed by restriction test digestion, see Figure 2) clones were then retransformed into yeast cells and reidentified in their groove protein-interaction potential. This procedure led to a total yield of 56 positive reactions for pUL50-screened clones, 11 for BFRF1-screened clones and 0 for VZV Orf24-screened clones (Table 1, lower three lines; see Figure S1 for the entire series of primary screening data with Lib1/pUL50).

A very similar screening round was performed with Lib2, as analyzed for interaction with non-autologous BFRF1 (Table 2). In this case the rate of retransformation/ reidentification led to a yield of 11 positive reactions for BFRF1-screened clones. The findings of the screenings with Lib1 and Lib2 thus revealed a number of valuable clones with specific interaction properties of interest, and thereby provided the first proof of this novel concept. Even when considering the Y2H system as a typical screening model, which is limited by the lack of a virological context, these data provided very relevant information. They illustrated that the aspired shared-hook binding properties, exerted by mutant hook constructs, were not found as a rare event, but actually occurred repeatedly by the analysis of both libraries used. Hereby, the increased substitution average of 3.4 in Lib2 had only minor effect on the yield of positive clones.

**Figure 6:**
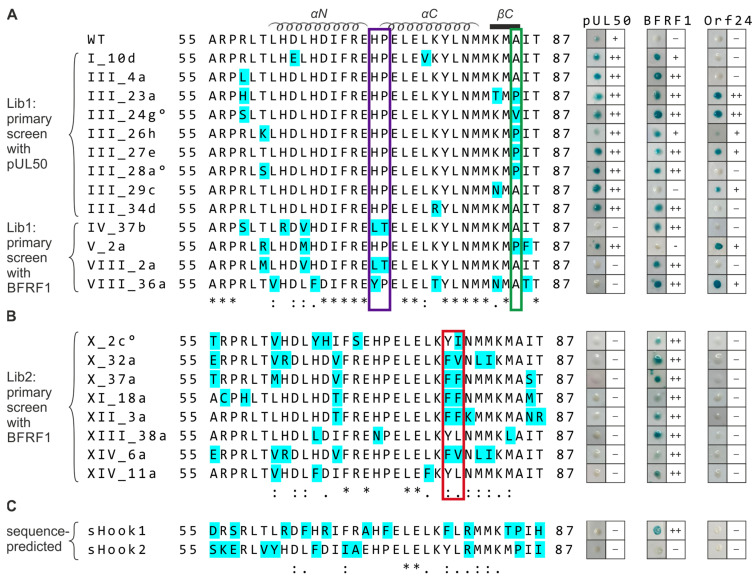
Sequence alignment and Y2H data comparing the hook region of WT pUL53, library-selected and sequence-predicted clones referring to their shared-hook binding properties with three different groove proteins. Amino acid sequences of the (**A**,**B**) WT pUL53 hook region (amino acids 55–87), the library clones listed below and (**C**) the sequence-predicted shared-hook constructs were aligned using the online multiple sequence alignment tool ClustalW2. Mutated residues are highlighted in cyan. °, indicates further amino acid exchanges were detected upstream the hook region (i.e., A43T for III_24g, R18S for III_28a and R8C for X_2c); *, positions of fully conserved residues; :, positions conserved within groups of strongly similar properties (i.e., scoring > 0.5 in the Gonnet PAM 250 matrix); ., positions conserved within groups of weakly similar properties (i.e., scoring ≤ 0.5 and >0 in the Gonnet PAM 250 matrix). In the Y2H analysis, pUL53 library clones were identified by primary screening against HCMV pUL50 (**A**, upper part) or EBV BFRF1 (**A**, lower part, **B**). All primary positive clones, including sequence-predicted constructs (**C**, sHook1 and sHook2), were then used for a backtransformation-based confirmation step and shared-hook analysis against HCMV pUL50, EBV BFRF1, or VZV Orf24, respectively, as shown at the right. The positivity rate of blue yeast colonies detected in the Xgal filter lift assay is indicated: ++, ≥50%; +, <50%; –, not detectable.

Interestingly, also the sequence-predicted constructs showed partial positive reaction when similarly assayed in the Y2H system (Figure 6). An experiment performed to illustrate the reactivity of these full-length constructs of hybrid hooks demonstrated a positive signal for sHook1 against BFRF1 groove, albeit negative against pUL50 and Orf24. The second construct, sHook2, behaved differently and remained negative against the three analyzed groove constructs (Figure 6C). A comparative alignment of clone sequences and Y2H data summarizes these findings for the hook region of WT pUL53 (Figure 6A), library-selected (Figure 6A,B) and sequence-predicted clones (Figure 6C) referring to their shared-hook binding properties with three different groove proteins. The sequence information points to the importance of amino acid position alanine 85 (Figure 6A). Four clones with triple shared-hook activity against the three analyzed groove proteins of α-, β-, and γ-herpesviruses, i.e., VZV Orf24, HCMV pUL50 and EBV BFRF1, possess an amino acid exchange at residue A85 in the βC-fold of the pUL53 hook structure (A85P or A85V; Figure 6A, green box). This implicates that this mutation site in the ßC-sheet may contribute to the shared-hook activity. Except for HCMV, all hook proteins shown in Figure 3 exhibit a proline as wild-type residue at position 85. This also applies to the hook protein of MCMV, which can bind in a nonautologous fashion to the HCMV groove [17,20]. This observation suggests that proline is generally tolerated at position 85 in herpesviral hook proteins underscoring the importance of this position for a broadened binding specificity.

The Lib1 screening (Figure 6A) also reveals sequence positions highly important for the HCMV hook-into-groove interaction. Most prominently, all hook sequences that lost HCMV groove binding, show changes at position H71 and/or P72 (Figure 6A, purple box). This finding suggests that these residues are important for stabilizing the link between the αN and αC helix of the hook. The proline is conserved in α- and β-herpesviruses but not in γ-herpesviruses (Figure 3). This explains the observation that Lib1-derived hook mutants, which show a mutation of P72, are still able to interact with BFRF1 (Figure 6A). However, the data referring to Lib2-derived hook mutants shows that conservation of the H71/P72-specific motif alone is not sufficient for pUL50 binding. This motif is seen conserved in almost all Lib2-derived hook mutants, also in those that do not bind to pUL50 (Figure 6B). One explanation for this unexpected finding may be a loss of binding affinity due to the strong variation of sequence positions 78 and 79 (Figure 6B, red box), which are highly important for the pUL53–pUL50 interaction. Replacement of either Y78 or L79 by alanine was shown to weaken the pUL53–pUL50 interaction by more than two orders of magnitude [17]. This suggests that replacements at this position, as detected in the Lib2-derived hook mutants, may disrupt a hot-spot of the HCMV hook–groove recognition. It should be generally emphasized that the gain in pUL53–BFRF1 binding is more difficult to rationalize than to a loss of pUL53–pU50 interaction. The hook sequences that are compatible with pUL53–BFRF1 interaction differ both in the altered sequence positions and the types of amino acid exchange observed. Due to the complexity of the mutation patterns observed, an understanding of BFRF1 binding specificity will require the experimental determination of the structure of groove protein BFRF1 in complex with selected shared-hook variants. Such a structural determination may also answer the question whether a local structure adjustment of the BFRF1 groove is sufficient to accommodate these shared-hook bindings, or whether larger structural rearrangements of the hook and/or BFRF1 groove sequences may be required to allow for interaction.

### 3.3. Verification of Shared-Hook Constructs by Coimmunoprecipitation (CoIP) Experiments

#### 3.3.1. Level of Confirmatory CoIP Analysis for Y2H Library Clones

**Figure 7:**
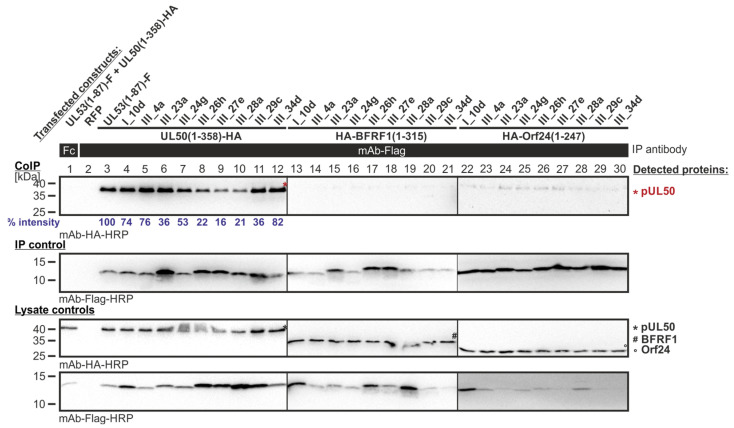
CoIP-based interaction analysis of HCMV pUL50, EBV BFRF1 and VZV Orf24 with the library clones that exerted shared-hook binding activity in the primary screening of Lib1 with pUL50. 293T cells were transiently transfected with plasmids coding for the HA-tagged groove proteins, pUL50, BFRF1 or Orf24, lacking their TMD, in combination with the Flag-tagged library constructs. The wild-type interaction of truncated pUL53 with pUL50 served as a positive and RFP as a negative control. At three d p.t., cells were lysed and Flag-tagged proteins were immunoprecipitated using mAb-Flag; a chicken Fc fragment served as a specificity control. Lysate controls taken prior to the IP and CoIP samples were subjected to standard Wb analysis using tag-specific antibodies as indicated. Blue numbers: each individual CoIP band was first normalized to the corresponding IP signal and was then set in relation to the reference WT interaction (% intensity) using Aida Image Analyzer v.4.23 software (mean values of triplicate densitometric determinations are given); CoIP was performed once.

In a first setting of CoIP experimentation, those Y2H-selected library clones were analyzed that resulted from the primary screening of Lib1 with pUL50 as the bait. All of the clones produced a strong CoIP signal for autologous pUL50 interaction (Figure 7, lanes 4–12), but lacked signals for nonautologous interactions with BFRF1 (lanes 13–21) or Orf24 (lanes 22–30; note that the slight bands seen in lanes 24–27 were considered as background signals as they do not represent the molecular size of Orf24, i.e., compare with lysate controls showing the protein input below). The interaction of the parental pair pUL50–pUL53 (lane 3) was used as a positive control; an RFP sample and lack of IP with a nonreactive antibody Fc fragment served as negative controls (lanes 1–2). Thus, the result indicated that this first series of library clones, which showed shared-hook binding activity in the Y2H system, could not be verified by CoIP in the human cell system.

**Figure 8:**
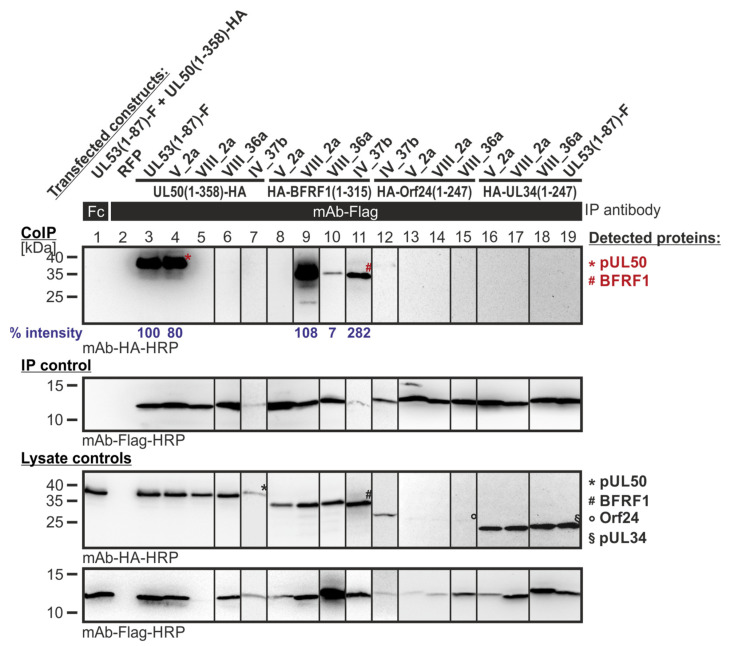
CoIP-based interaction analysis of HCMV pUL50, EBV BFRF1, VZV Orf24 and HSV-1 pUL34 with the library clones that exerted shared-hook binding activity in the primary screening of Lib1 with BFRF1. 293T cells were transiently transfected with plasmids coding for the HA-tagged groove proteins, pUL50, BFRF1, Orf24, or pUL34, lacking their TMD, in combination with the Flag-tagged library constructs. The wild-type interaction of truncated pUL53 with pUL50 served as a positive and RFP as a negative control. At three d p.t., cells were lysed and Flag-tagged proteins were immunoprecipitated using mAb-Flag; a chicken Fc fragment served as a specificity control. Lysate controls taken prior to the IP and CoIP samples were subjected to standard Wb analysis using tag-specific antibodies as indicated. Blue numbers: each individual CoIP band was first normalized to the corresponding IP signal and was then set in relation to the reference WT interaction (% intensity) using Aida Image Analyzer v.4.23 software (mean values of triplicate densitometric determinations are given); CoIP was performed once.

In a second setting of CoIP experimentation, those Y2H-selected library clones were analyzed that resulted from the primary screening of Lib1 with BFRF1 as the bait. Hereby, the BFRF1 reactivity could be verified in the CoIP system, showing strong or intermediate signal strengths, for three clones (Figure 8, lanes 9, 10, and 11, respectively), while clone V_2a remained negative (lane 8). Most interestingly, the three BFRF1-positive mutants of the pUL53 hook, i.e., clones VIII_2a, VIII_36a and IV_37b (that exerted a CoIP interaction with BFRF1; lanes 9, 10, and 11), lost their interaction potential with the autologous pUL50 (lanes 5, 6 and 7). In particular, the first mentioned clone, VIII_2a, was highly BFRF1-reactive, comparable with the CoIP positive control (lane 3). This finding indicates that the Y2H screening of pUL53 hook mutants directed to the BFRF1 bait resulted in the identification of three examples of ‘changed-hook activity’ (nonautologous BFRF1 positive, but autologous pUL50 negative).

**Figure 9:**
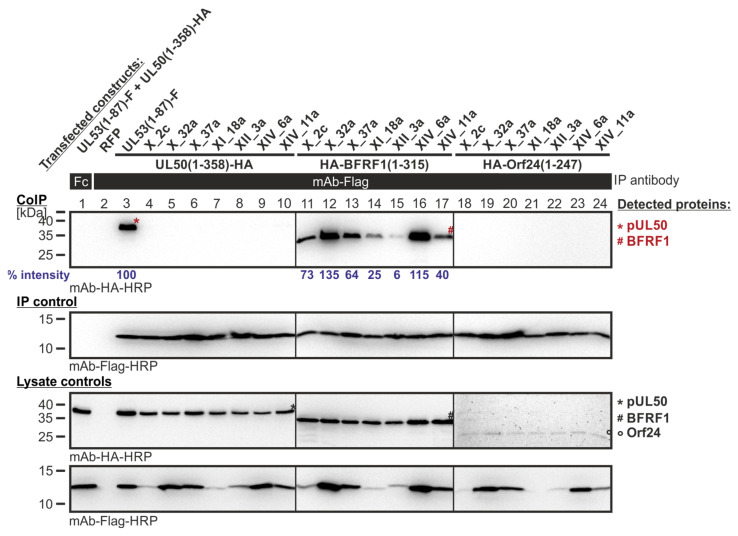
CoIP-based interaction analysis of HCMV pUL50, EBV BFRF1 and VZV Orf24 with the library clones that exerted shared-hook binding activity in the primary screening of Lib2 with BFRF1. 293T cells were transiently transfected with plasmids coding for the HA-tagged groove proteins, pUL50, BFRF1, or Orf24, lacking their TMD, in combination with the Flag-tagged library constructs. The wild-type interaction of truncated pUL53 with pUL50 served as a positive and RFP as a negative control. At three d p.t., cells were lysed and Flag-tagged proteins were immunoprecipitated using mAb-Flag; a chicken Fc fragment served as a specificity control. Lysate controls taken prior to the IP and CoIP samples were subjected to standard Wb analysis using tag-specific antibodies as indicated. Blue numbers: each individual CoIP band was first normalized to the corresponding IP signal and was then set in relation to the reference WT interaction (% intensity) using Aida Image Analyzer v.4.23 software (mean values of triplicate densitometric determinations are given); CoIP was performed once.

In an additional approach, those Y2H-selected library clones were analyzed that resulted from the primary screening of Lib2 with BFRF1 as the bait. The data of this CoIP experimentation confirmed the observed interactions of the Y2H analysis. All analyzed clones showed CoIP signals for the BFRF1 interaction (Figure 9, lanes 11–17). The signal strength for the CoIP interaction varied within the tested library clones. Clone XII_3a (lane 15) showed a rather weak band, while clones X_2c, XI_18a and XIV_11a (lanes 11, 14, 17) produced an intermediate band and clones X_32a, X_37a and XIV_6a (lanes 12, 13, 16) resulted in a strong band for BFRF1 interaction. Remarkably, all seven clones also lost the property to interact with the parental pUL50 and hence bind exclusively BFRF1, i.e., comprise changed hook activity. 

#### 3.3.2. Level of Confirmatory CoIP Analysis for Sequence-Predicted Hybrid Constructs

**Figure 10:**
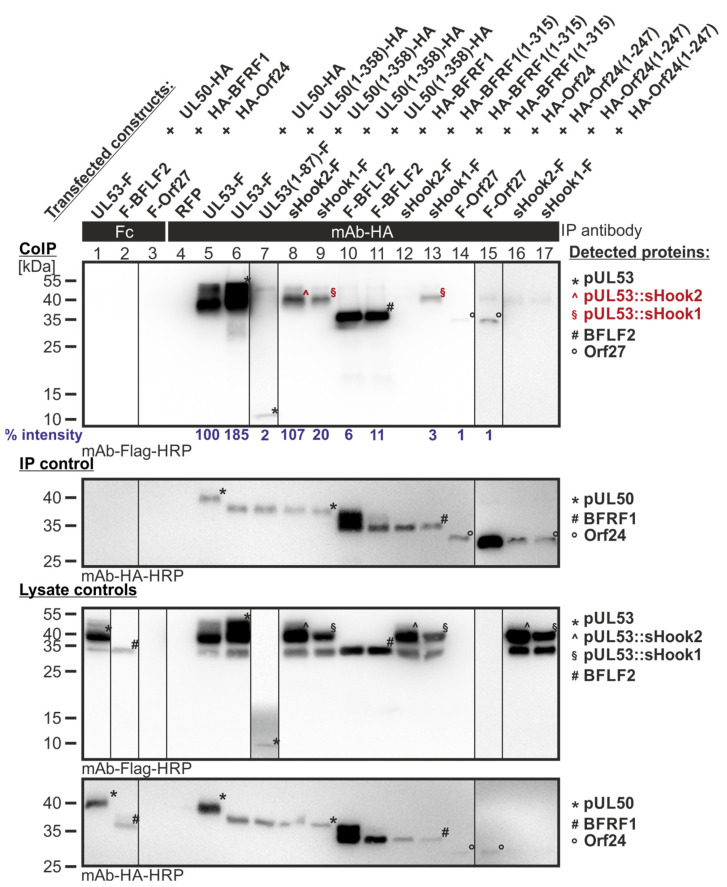
CoIP-based interaction analysis of sequence-predicted shared-hook constructs with three groove proteins. 293T cells were transiently transfected with constructs coding for the HA-tagged groove proteins, pUL50, BFRF1 or Orf24, either full-length or without TMD, in combination with the Flag-tagged sequence-predicted shared-hook constructs. Positive controls were the original hook proteins pUL53, BFLF2 or Orf27 and RFP served as a negative control. At three d p.t., cells were lysed and HA-tagged proteins were immunoprecipitated using mAb-HA; a chicken Fc fragment served as a specificity control. Lysate controls taken prior to the IP and CoIP samples were subjected to standard Wb analysis using tag-specific antibodies as indicated. Interaction of the sequence-predicted shared-hook constructs is indicated with red symbols. Blue numbers: each individual CoIP band was first normalized to the corresponding IP signal and was then set in relation to the reference WT interaction (% intensity) using Aida Image Analyzer v.4.23 software (mean values of triplicate densitometric determinations are given); CoIP was performed three times.

Next, also the sequence-predicted hybrid constructs were analyzed by confirmatory CoIP experimentation. Control panels indicated that the constructs were expressed properly (Figure 10, lysate controls) and that IP using the mAb-HA was positive for all three groove proteins of interest, i.e., HCMV pUL50, EBV BFRF1 and VZV Orf24 (Figure 10, IP controls). The autologous NEC pairs, used as reference positive controls, showed reliable CoIP signals, in strong, intermediate or weak signal strengths, respectively, for the three viral NECs analyzed (lanes 5–6, 10–11, 14–15; note that all three groove proteins were expressed as full-length versions, as well as truncated versions lacking their transmembrane anchors, likewise used in the Y2H settings). As an important finding, both hybrid constructs of the HCMV hook protein pUL53, i.e., sHook1 (EBV BFLF2-adapted) and sHook2 (VZV Orf27-adapted), showed a pronounced interaction with the HCMV groove construct pUL50(1–358)-HA (lanes 8–9). Even more valuable is the finding that sHook1 also showed, at somewhat lower signal strength, an interaction with the EBV groove construct HA-BFRF1(1–315) (lane 13). This indicated a shared-hook binding activity towards the HCMV- and EBV-specific groove proteins for sHook1, while reactivities with VZV Orf24 were negative (lanes 16–17). Combined, the CoIP experiments performed with the various clones and constructs derived from the two different approaches (i.e., mutagenesis libraries and sequence-predicted) confirmed our concept: shared-hook binding activity can be achieved through specific replacement adaptations in the NEC hook element.

### 3.4. Verification of Shared-Hook Constructs by Confocal Imaging Experiments

**Figure 11:**
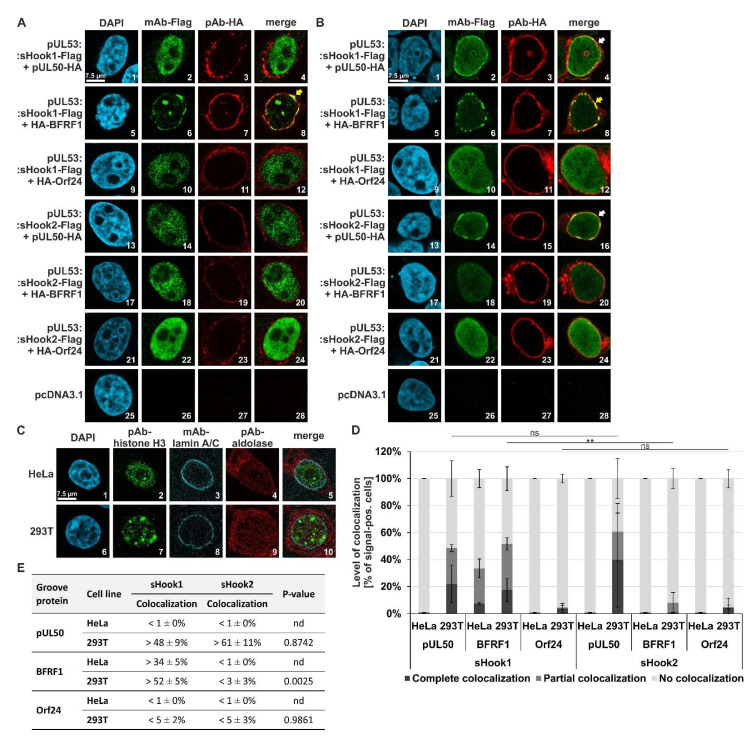
Qualitative and quantitative analyses of nuclear rim colocalization patterns: coexpressed sequence-predicted hybrid constructs (HCMV/EBV pUL53 sHook1 and HCMV/VZV pUL53 sHook2) in combination with three different groove proteins (HCMV pUL50, EBV BFRF1 or VZV Orf24). (**A**) HeLa or (**B**) 293T cells were transiently cotransfected with either constructs coding for the Flag-tagged sequence-predicted hooks, sHook1 or sHook2, and the HA-tagged groove proteins pUL50, BFRF1 or Orf24. Two d p.t., cells were fixed, used for immunostaining with tag-specific antibodies and analyzed by confocal imaging. DAPI counterstaining indicated the morphology of nuclei of the respective cells. In both types of cells, the localization patterns of coexpressed construct pairs were analyzed; the scale bar in panels A–C, picture 1 marks 7.5 μm. On a qualitative level of analysis (**A**,**B**), the yellow arrows indicate complete nuclear rim colocalization, and the white arrows show partial rim colocalization. (**C**) Staining of subcellular localizations of marker proteins in HeLa and 293T cells, i.e., the nuclear lamina, the nucleoplasmic and cytosolic compartments. (**D**) Quantitation of the colocalization patterns of pUL53 sHook1 and sHook2 with groove proteins HCMV pUL50, EBV BFRF1 and VZV Orf24. Localization patterns observed in HeLa and 293T cells were quantified and classified as complete, partial or no colocalization. Mean values ± SD are given as expressed in a percentage of the entire number of counted cells. Statistical analysis was performed using an ordinary two-way ANOVA and post-hoc Tukey correction; **, *p* < 0.01; ns, not significant. (**E**) Summarized findings of colocalization obtained for the two sequence-predicted hybrid constructs, combining complete and partial colocalization as total percentages; nd, not determined.

As an additional step of confirmation of interaction patterns in human cell systems, a confocal imaging-based assay of nuclear rim colocalization was performed under methodological conditions published earlier [19,20,34,36,37,38,39]. This assay was geared to determine the shared-hook interaction capacity, measured by a putative nuclear rim colocalization on the single-cell level, between the two sequence-predicted hook hybrid constructs and either of three different, coexpressed herpesviral groove proteins (Figure 11). On a qualitative level, representative images were scored to visualize the detected patterns for all combinations (Figure 11, panel A for Hela cells, panel B for 293T cells). The autologous control pair of pUL50–pUL53, routinely used a reference control, showed a complete nuclear rim signal of perfect colocalization in double-positive cells (data not shown here; [19]), and vector controls were devoid of background signals (panels A and B, images 25–28). Note the patterns of perfect and partial nuclear rim colocalization marked by yellow or white arrows, respectively (panel A, image 8; panel B, images 4, 8 and 16; note, that perfect nuclear rim colocalization was defined as a very distinct rim localization of pUL53, with a strong reduction of diffuse signals remaining in the nucleoplasm). The assignment of individual proteins to intracellular localization patterns was verified by staining of cellular marker proteins (Figure 11C). These data point to a confirmed interaction between sHook1 with pUL50 and BFRF1, as well as sHook2 with pUL50. On the quantitative level, values express a more pronounced pattern of colocalizations found for cotransfected 293T cells (Figure 11B) than HeLa cells (Figure 11A), a finding that might refer to higher intranuclear coexpression levels of these constructs in individual 293T cells. In essence, the HCMV pUL53 hybrid construct sHook1 (EBV BFLF2-adapted) indicated an activity of shared-hook binding activity through its colocalization with both pUL50 and BFRF1 (Figure 11D, left part). In comparison, the hybrid construct sHook2 (VZV Orf27-adapted) also showed clear pUL50 colocalization, but only limited BFRF1 colocalization. Interestingly, in this assay construct sHook2 additionally showed some tendency of VZV Orf24 colocalization at least in 293T cells (Figure 11D,E, <5%), which was not detected by CoIP analysis but was compatible with the idea of VZV-specific adaptation of this pUL53 hybrid construct. Expression levels for the two constructs were determined by microscopic countings of signal-positive cells (mean values ± SD; Table S2). Summarized, the quantities of positive nuclear rim colocalization were specifically pronounced in 293T cells showing values of >48 ± 9% (pUL50) and > 52 ± 5% (BFRF1) for sHook1, as well as > 61 ± 11% (pUL50) for sHook2 (Figure 11E). Thus, the confocal imaging data are compatible with the CoIP findings described above.

### 3.5. Complementation Analysis Using Recombinant HCMV AD169-GFP ΔUL53 for the Infection of HFF Populations Conditionally Expressing pUL53 Mutants

**Figure 12:**
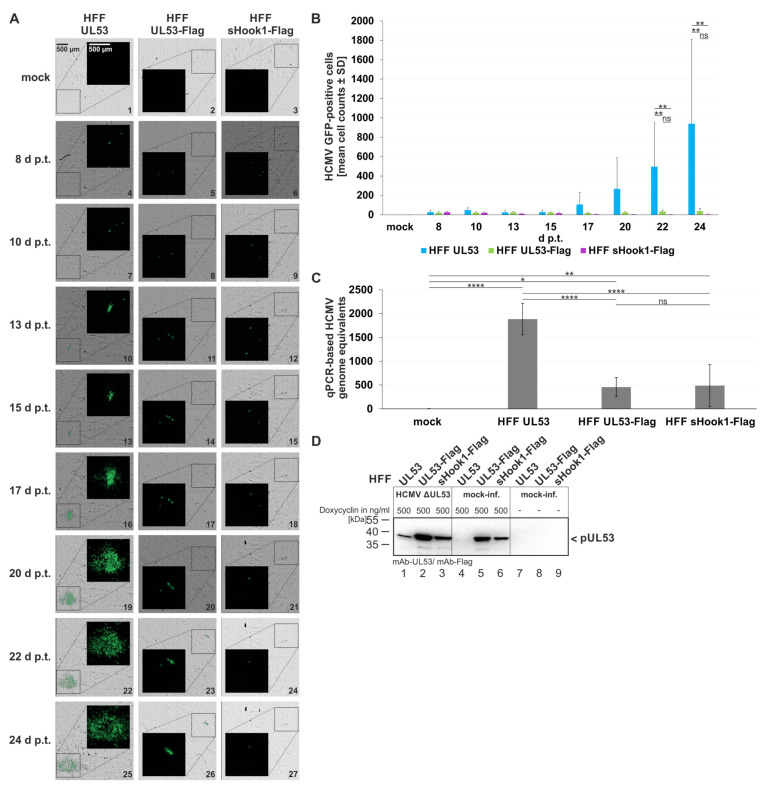
Qualitative and quantitative analyses of HCMV AD169-GFP ΔUL53 virus reconstitution on the different recombinant HFF cell populations. HFFs that express pUL53, pUL53-Flag or pUL53::sHook1-Flag were transfected with the infectious bacterial artificial chromosome of HCMV ΔUL53. (**A**) Images of the GFP signals and the respective brightfield illuminations were taken at indicated time points; scale bar in panel A, picture 1 marks 500 μm. (**B**) Quantitative analysis of fluorescence-positive cells was achieved with automated counting by the CellReporterXpress^®^ software (Molecular Devices LLC, San Jose, CA, USA). Counting parameters for positive nuclei were set to an intensity of at least 100, a minimal width of 7 and a maximal width of 30. Measurements were performed in biological sextuplicates per cell population of 5.05% area of the well and mean values ± SD are given. (**C**) qPCR-based assay for the determination of viral genome equivalents referring to the respective recombinant HFF populations. Viral supernatants were harvested at day 24 p.t. and subjected to IE1-specific qPCR. Calculations were performed in biological sextuplicates per cell population; mean values ± SD are shown. (**B**,**C**) Statistical analysis was performed using an ordinary two-way ANOVA and post-hoc Tukey correction; ****, *p* < 0.0001; **, *p* < 0.01; *, *p* < 0.1; ns, not significant; no significant differences were noted for values between 1–20 d p.t. (**B**). (**D**) Wb-based expression analysis of the conditionally expressing HFF populations. Recombinant protein expression in the three HFF populations of HFF-UL53, HFF-UL53-Flag and HFF-UL53::sHook1-Flag cells was either uninduced (-) or induced (500 ng/mL Dox). Cells were either infected with HCMV ΔUL53 or remained mock-infected. At 24 h p.t., cells were harvested and lysed. Total lysate samples were subjected to standard Wb analysis using tag-specific or protein-specific monoclonal antibodies as indicated.

In order to investigate the intact functionality of conditionally expressed pUL53 in selected HFF populations, an infection complementation analysis was performed. To this end, HFFs that express pUL53, pUL53-Flag or pUL53::sHook1-Flag were used for BAC transfection using a genomic DNA preparation of the construct ΔUL53. The transfected cells were steadily supplemented with inducer medium containing 500 ng/mL of Dox in order to ensure the constant expression of one of the three recombinant versions of pUL53. In a time schedule up to 24 d p.t., cell samples were subjected to ImageXpress^®^ Pico-based green fluorescent protein (GFP) microscopy for depicting the reporter expression of GFP-encoding HCMV ΔUL53 (Figure 12A). As an important finding, GFP-positive cells were produced in all three settings of infection, thereby indicating that on a single-cell basis the pUL53 wild-type (HFF UL53), the Flag-tagged version (HFF UL53-Flag) and also the shared-hook version (HFF sHook1-Flag) showed functionality on complementing the viral ΔUL53 defect (Figure 12A, see insets with GFP-positive areas increasing over time). A quantitative evaluation of GFP signals (Figure 12B) underlined this microscopic observation. The consecutive increase of GFP signals was mostly seen with HFF UL53, while the other two UL53 versions remained at a limited level. For this reason, virus reconstitution and the production of a stock of HCMV ΔUL53 was specifically done with the HFF UL53 setting. Nevertheless, the presence of HCMV genome equivalents, derived from the release of infectious virus into the supernatant of the cell populations, could be demonstrated by HCMV-specific qPCR with supernatant samples of all three settings (Figure 12C). Likewise, an analysis of pUL53 production in ΔUL53-infected, complementing cells indicated the increase of pUL53 as a consequence of infection with HCMV ΔUL53 (Figure 12D, lanes 1–3) compared to mock-infected cells (lanes 4–6) or uninduced/mock-infected control cells (lanes 7–9). This increase of pUL53 is explained by the presence of the HCMV immediate early promoter region contained within the Dox-inducible module responsible for recombinant expression [23]. These results strongly argue for the functionally intact nature of the complementing pUL53, providing evidence for fully supporting HCMV ΔUL53 replication in lytically infected cells.

**Figure 13:**
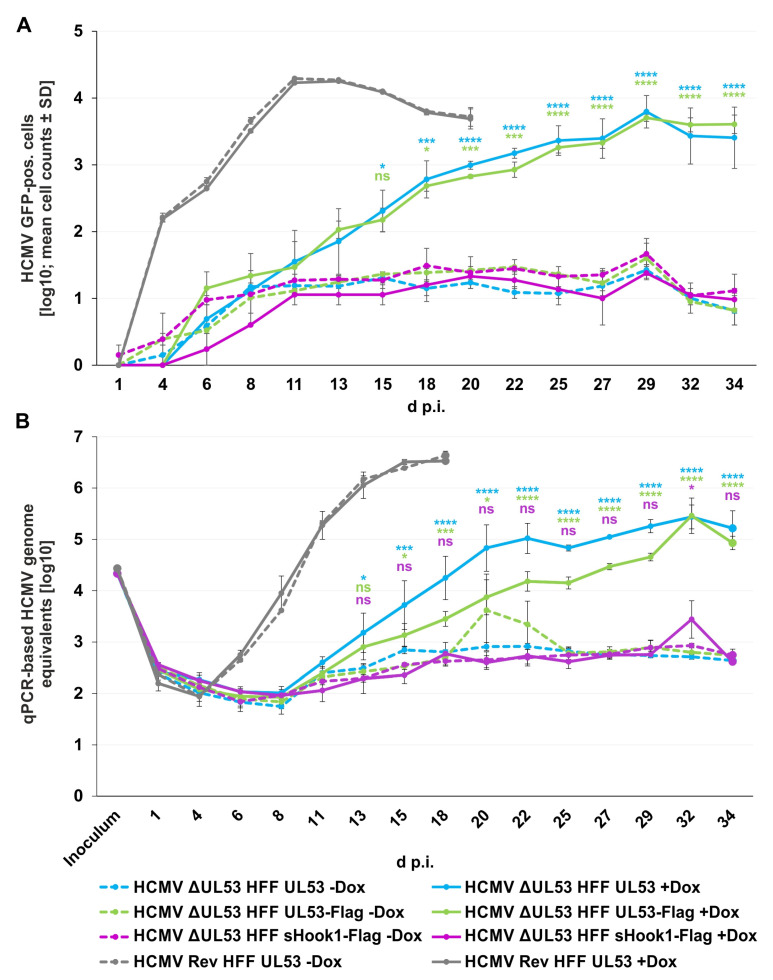
Viral replication kinetics of HCMV ΔUL53 and its revertant (HCMV Rev) determined by quantitation of GFP-positive cells and HCMV-specific qPCR on the different recombinant HFF populations. 80,000 inducibly expressing HFFs in 24-well plates were infected with HCMV ΔUL53 or HCMV Rev at a viral dose of 5 × 10^6^ genome copies. pUL53, pUL53-Flag or pUL53::sHook1-Flag protein expression was either Dox-induced (+Dox) or remained non-induced (−Dox). (**A**) The number of HCMV-infected cells was measured by detection of GFP signal-positive cells at indicated time points with the CellReporterXpress^®^ software using the ImageXpress^®^ Pico device. Values represent 25.04 % of the area of a well and are given as a mean value ± SD of two independently infected wells. (**B**) Viral supernatants were harvested at indicated time points and viral genome equivalents were determined by qPCR. Each value represents the mean ± SD of two independent biological replicates, each measured twice. (**A**,**B**) Statistical analysis was performed using an ordinary two-way ANOVA and post-hoc Sidak correction; ****, *p* < 0.0001; ***, *p* < 0.001; *, *p* < 0.1; ns, not significant; no significant differences were noted for values between 1–13 d p.i. (**A**) or 1–11 d p.i. (**B**), respectively.

Finally, the question was addressed whether pUL53 wild-type compared to the versions pUL53-Flag and sHook1-Flag similarly exert the activity of ΔUL53 complementation. To this end, viral replication kinetics were performed and evaluated through two different methodological readouts. Over a period of 34 days post-infection (d p.i.), the quantities of virus-positive cells were analyzed for the various complementation settings in parallel, both in the presence (+Dox) or absence of doxycycline (−Dox; Figure 13). By the analysis of the GFP-positive cells (Figure 13A) and supernatant of infected cells (Figure 13B), the kinetics of viral replication was monitored. Both, HFF UL53 and HFF UL53-Flag showed a steady increase of viral replication (+Dox), i.e., HCMV ΔUL53 replication was supported by these complementing cells (Figure 13A,B, blue and green solid curves). In the absence of Dox (−Dox), replication remained very low at a background level (Figure 13A,B, blue and green dashed curves). Interestingly, the expression of sHook1-Flag (+Dox) in HFFs seemed not to support HCMV ΔUL53 propagation. Only a minor peak of viral signals (29–34 d) was observed, but remained within the range of uninduced (−Dox) signals (Figure 13A,B, purple solid and dashed curves). In comparison, HCMV Rev comprised a maximal level of viral replication in HFF UL53 cells, independent from +Dox or −Dox conditions (Figure 13A,B, grey solid and dashed curves). Regarding the kinetics of HCMV Rev, the kinetics of HCMV ΔUL53 in HFF UL53 and HFF UL53-Flag cells revealed some reduction in replication efficiency, which may reflect the difference between wild-type-like viral replication conditions and the complementation system, respectively. In addition, the second replicate of this experiment was performed, by increasing the viral absorption time (without a removal of the viral inoculum; Figure S2), thus confirming the finding pictured in Figure 13. These findings indicate that pUL53 and pUL53-Flag are capable of functional ΔUL53 complementation, whereas pUL53::sHook1-Flag appeared to be restricted in this activity.

## 4. Discussion

With this study, we aimed to identify a common structural panherpesviral NEC scaffold that enables hook-into-groove interactions between NEC proteins derived from members of different viral subfamilies. Especially, the question was addressed whether experimental mutagenesis of a given herpesviral NEC hook protein (in our case HCMV pUL53) may adapt its binding specificity to more than one herpesviral NEC groove protein (α-, β- or γ-herpesviral). This theoretical option was thus termed shared-hook binding activity, and we applied two screening approaches, i.e., the library-based random mutagenesis and the sequence-predicted generation of specific hybrid hook constructs. The combined findings of this study are as follows: (i) series of mutant/recombinant hook constructs were generated, either in the form of random libraries or sequence-predicted hybrid constructs, for analyzing their binding activities; (ii) the screening levels revealed a number of cross-viral, shared hook-into-groove binding clones within the Y2H system; (iii) interestingly, on the level of human-cell expression, such shared-hook binding property could exclusively be reproduced positive for one of the sequence-predicted hybrids, sHook1, while randomized library mutants were comparably negative; other randomized Y2H mutants, however, showed a reproducible tendency to generate a changed-hook binding property, thus interacting with nonautologous groove partners also in the human-cell system; and finally (iv) the pUL53 complementation analysis of HCMV ΔUL53 infection provided a strong indication that wild-type pUL53 and also a Flag-tagged version are able to functionally complement viral ORF UL53 depletion, whereas such complementing activity was very limited or even missing for the sHook1 hybrid.

In the comparison of the two approaches, the library-based approach, primarily investigated by Y2H screening rounds, yielded a number of mutagenized clones of pUL53 that exerted shared-hook binding activity in the yeast system. In addition, some of the library-derived mutants also showed an altered binding specificity towards a nonautologous groove protein. This important finding of the library-based screening led to the conclusion that distinct hook mutants have lost the property to interact with the parental NEC groove-binding partner, but acquired binding activity towards a different herpesviral groove, then termed as changed-hook binding activity. Even more pronounced, referring to the second approach, the sequence-predicted sHook1 hybrid construct exhibited a broadened binding characteristic, including more than one herpesviral groove protein (shared-hook binding including HCMV and EBV groove proteins). The latter aspect was specifically addressed by the respective analyses of CoIP, confocal nuclear rim colocalization and complementation experiments with HCMV ΔUL53 in cell populations expressing pUL53 mutants. Concludingly, this sHook1 hybrid showed such true shared-hook binding activity in human cells. Nevertheless, the current data of the functional complementation analysis with pUL53-expressing cells strongly pointed to the notion that a proven hook binding activity does not necessarily mean full functionality in nuclear egress and viral replication. This was illustrated by the finding that the sHook1 hybrid, despite comprising strong and broadened groove binding properties, showed no detectable complementation during HCMV ΔUL53 infection.

Related studies of other researchers are illustrating these results on the importance of core NEC interaction on a wider basis of herpesvirus infections. For HSV-1 and pseudorabies virus (PrV), a complete deletion of ORF UL31 led to strongly decreased viral loads, which could be restored by the use of complementing cells [40,41,42]. A very similar finding was also described for the deletion of ORF BFLF2, the homolog of EBV. In this study, the defective phenotype was restored by trans-complementation with a BFLF2 expression plasmid [43]. Another study provided additional insight into the cytomegaloviral NEC by using the murine CMV homologs [44]. The null phenotype of the M53-deleted virus was rescued by a reintroduction of the ORF M53 at an ectopic position in the genome. In summary, these studies and our present results confirm the general functional importance of herpesviral core NEC proteins, i.e., data demonstrate that deletion of UL53, or homologs, leads to defective viral replication, which can be restored by complementation. 

Further studies, including our own work, addressed the possibility of cross-interactions and cross-complementation of the core NEC proteins belonging to different herpesviruses. In a transient transfection system, we could show that cross-interactions are restricted to core NEC proteins belonging to the members of the same herpesviral subfamilies [20]. A confirmation of this statement was provided by independent methods applied by several other researchers [45,46,47]. Until now, however, only a few studies investigated the true functional cross-complementation between these viral core NECs. Cross-complementation between human and murine CMVs was achieved through the genetic replacement of ORFs M50 or M53 by the respective HCMV homologous ORFs in the MCMV genome, thereby assuring functionality in terms of viral replication [47]. Moreover, such functional replacement of M50 by UL50 in the MCMV genetic background was also confirmed for the in vivo mouse model by our earlier study [20]. Interestingly, however, an analysis of the α-herpesviruses HSV-1 and VZV demonstrated that the replacement of UL34 by Orf24 did not yield fully replication-competent HSV-1. This was surprising, since on the protein level, positive signals for cross-viral HSV-1–VZV core NEC interaction were provided [48]. These studies demonstrate that cross-viral NEC interactions can be provided within members of one subfamily, but the phenotype of interaction may not be directly linked with full functional complementation. Taken together, as seen in the light of such earlier reports, our novel data specify the properties of herpesviral core NEC formation. The present findings render precisely the possibility to generate a hook region with the ability to bind to various viral groove proteins. Thus, it appears straightforward to utilize such information, obtained through the shared-hook binding model, for exploiting the multi-binding NEC mutants as prototype targets for the development of panherpesviral NEC-directed candidate drugs.

## 5. Conclusions

Our present report provides refined insight into the properties of cross-herpesviral core NEC formation and demonstrates that it is possible to generate a hook region with the ability to bind to various viral groove proteins or to acquire an altered binding property to a nonautologous hook. This statement was based on several independent experimental systems that included screening levels (Y2H), confirmation levels (human cells) and a ΔUL53 infection level (complementation). The main findings reach beyond our earlier state of knowledge, since previously, we and others favored the notion that core NEC interactions, as experimentally addressed in a cross-viral fashion, were found highly conserved within members of one of the α-/β-/γ-herpesviral subfamilies, such as core NEC interactions between HCMV–MCMV (β–β), HSV-1–VZV (α–α) or others. This view could now be further refined on the basis of novel data. The findings strongly support our statement that a shared phenotype of cross-viral core NEC interaction can be achieved through distinct mutations (i.e., random mutagenesis or sequence prediction-based hybrids), as introduced into the HCMV hook protein pUL53.

## Figures and Tables

**Table 1:** Screening procedure to identify pUL53 clones from Lib1 that exert shared-hook binding activity in the Y2H system.

	HCMV pUL53 Library (Lib1) Screened for Interaction with:		
	HCMV pUL50 # wt │ co	EBV BFRF1	VZV Orf24
Y153 cell number/0.5 µg library DNA	~2.87 × 10^7^	~3.64 × 10^7^	~3.49 × 10^7^
Interacting pUL53 library clones in primary screening *	20 │ 28	16	3
Recovered in *E. coli* DH10B ^§^	18 │ 27	16	2
Positive yeast retransformants with pUL50 *	17 │ 26	5	0
Positive yeast retransformants with BFRF1 *	8	4	0
Positive yeast retransformants with Orf24 *	5	2	0

* Filter lift assay measuring Xgal activity; ^§^ single clone growth in resistance selection media and test digestion for library DNA/hook construct; # first and second rounds of screening (using pUL50 wild-type (wt) or pUL50 codon-optimized (co), respectively).

**Table 2:** Screening procedure to identify pUL53 clones from Lib2 that exert shared-hook binding activity in the Y2H system.

	HCMV pUL53 Library (Lib2) Screened for Interaction with EBV BFRF1
Y153 cell number/0.5 µg library DNA	~5.09 × 10^7^
Interacting pUL53 library clones in primary screening *	35
Recovered in *E. coli* DH10B ^§^	31
Positive yeast retransformants with pUL50 *	2
Positive yeast retransformants with BFRF1 *	9
Positive yeast retransformants with Orf24 *	0

* Filter lift assay measuring Xgal activity; ^§^ single clone growth in resistance selection media and test digestion for library DNA/hook construct.

## Data Availability

The responsible authors declare that this article fully complies with the Data Availability Statements in section “MDPI Research Data Policies” at https://www.mdpi.com/ethics (accessed on 6 October 2022).

## Supplementary Materials

The following supporting information can be downloaded at: https://www.mdpi.com/article/10.3390/cells11244030/s1, Table S1: Oligonucleotide primers used in this study; Table S2: Protein expression levels of HeLa and 293T cells given a s mean values ± SD signal-positive cells; Figure S1: Sequence alignment: Y2H data comparing the hook region of WT pUL53 and library clones derived from the primary screening of Lib1 with HCMV pUL50 referring to their shared-hook binding properties with three different groove proteins; Figure S2: Viral replication kinetics of HCMV ΔUL53 and its revertant (HCMV Rev) determined by quantitation of GFP-positive cells and HCMV-specific qPCR on the different recombinant HFF populations.
