# Supplementary material for: ‘Shared-Hook’ and ‘Changed-Hook’ Binding Activities of Herpesviral Core Nuclear Egress Complexes Identified by Random Mutagenesis
Source: Cells. 2022 Dec 13;11(24):4030. doi: 10.3390/cells11244030 (PMC9776765; doi:10.3390/cells11244030)
Supplement: Supplementary file 1 [file cells-11-04030-s001.zip › cells-1986207-supplementary.pdf]

cells

MDPI

Supplementary materials

'Shared-hook' and 'changed-hook' binding activities of herpesviral core nuclear egress complexes identified by random mutagenesis

Josephine Lösing[1], Sigrun Häge[1], Martin Schütz[1], Sabrina Wagner[1], Julia Wardin[1], Heinrich Sticht[2] and Manfred Marschall[1,*]

1 Institute for Clinical and Molecular Virology, Friedrich-Alexander-Universität Erlangen-Nürnberg (FAU), 91054 Erlangen, Germany; josi.loesing@fau.de, sigrun.haege@fau.de, martin.schuetz@uk-erlangen.de, sabrina.wagner@uk-erlangen.de, julia.wardin@fau.de, manfred.marschall@fau.de

2 Division of Bioinformatics, Institute of Biochemistry, Friedrich-Alexander-Universität Erlangen-Nürnberg (FAU), 91054 Erlangen, Germany, heinrich.sticht@fau.de

* Correspondence: manfred.marschall@fau.de, Tel.: +49-9131-85-26089

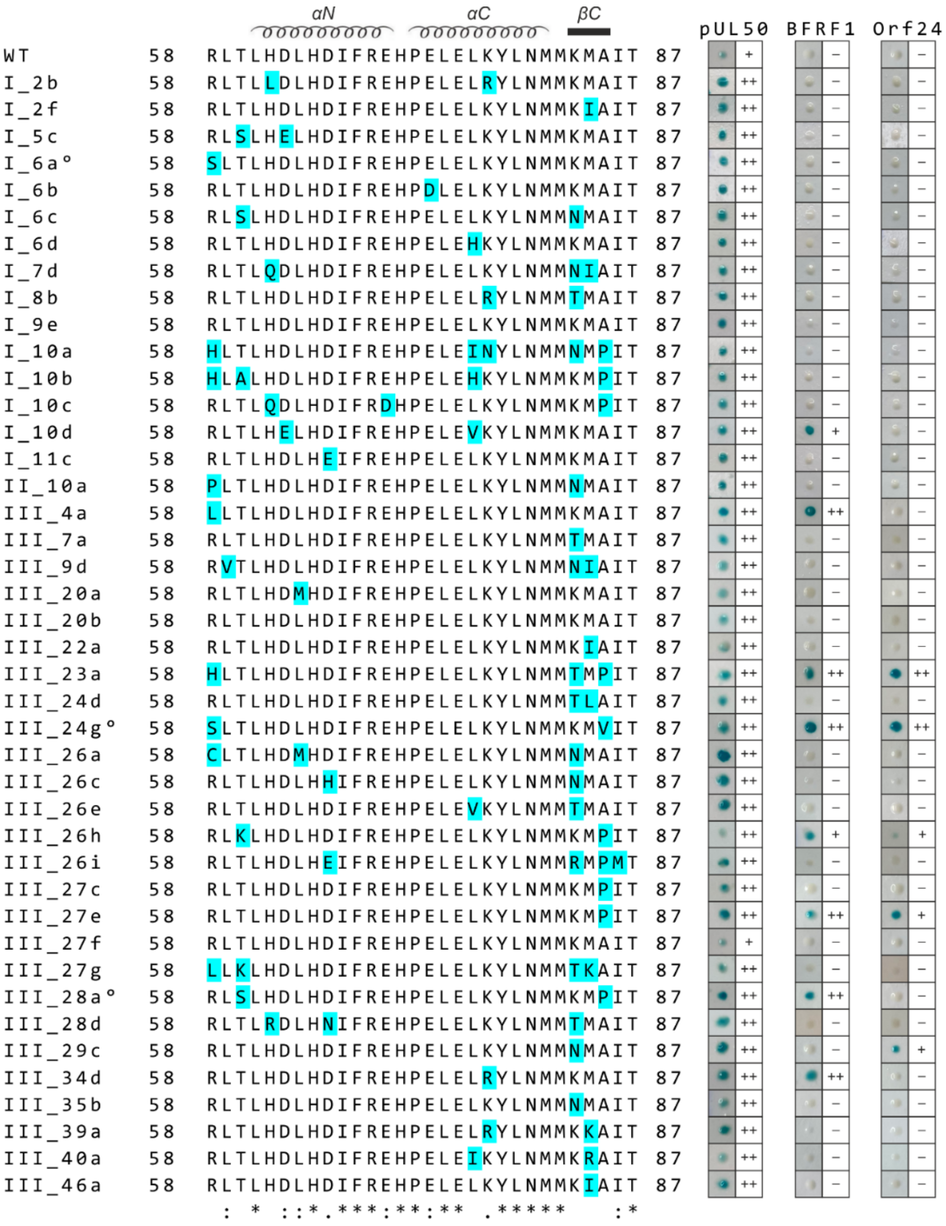

Figure S1. Sequence alignment: Y2H data comparing the hook region of WT pUL53 and library clones derived from the primary screening of Lib1 with HCMV pUL50 referring to their shared-hook binding properties with three different groove proteins. Amino acid sequences of the WT pUL53 hook region (amino acids 58- 87) and the library clones listed below were aligned using the online multiple sequence alignment tool ClustalW2. Mutated residues are highlighted in cyan. °, indicates further amino acid exchanges were detected upstream the hook region (i.e. A55T for clone I_6a, A43T for III_24g and R18S for III_28a); *, positions of fully conserved residues; :, positions conserved within groups of strongly similar properties (i.e. scoring > 0.5 in the Gonnet PAM 250 matrix); ., positions conserved within groups of weakly similar properties (i.e. scoring ⩽ 0.5 and > 0 in the Gonnet PAM 250 matrix). In the Y2H analysis, pUL53 library clones were identified by primary screening against HCMV pUL50. All primary positive clones were then used for a retransformation-based confirmation step and shared-hook analysis against HCMV pUL50, EBV BFRF1, or VZV Orf24, respectively, as shown at the right. The positivity rate of blue yeast colonies detected in the Xgal filter lift assay is indicated: ++, ⩾ 50%; +, < 50%; −, not detectable.

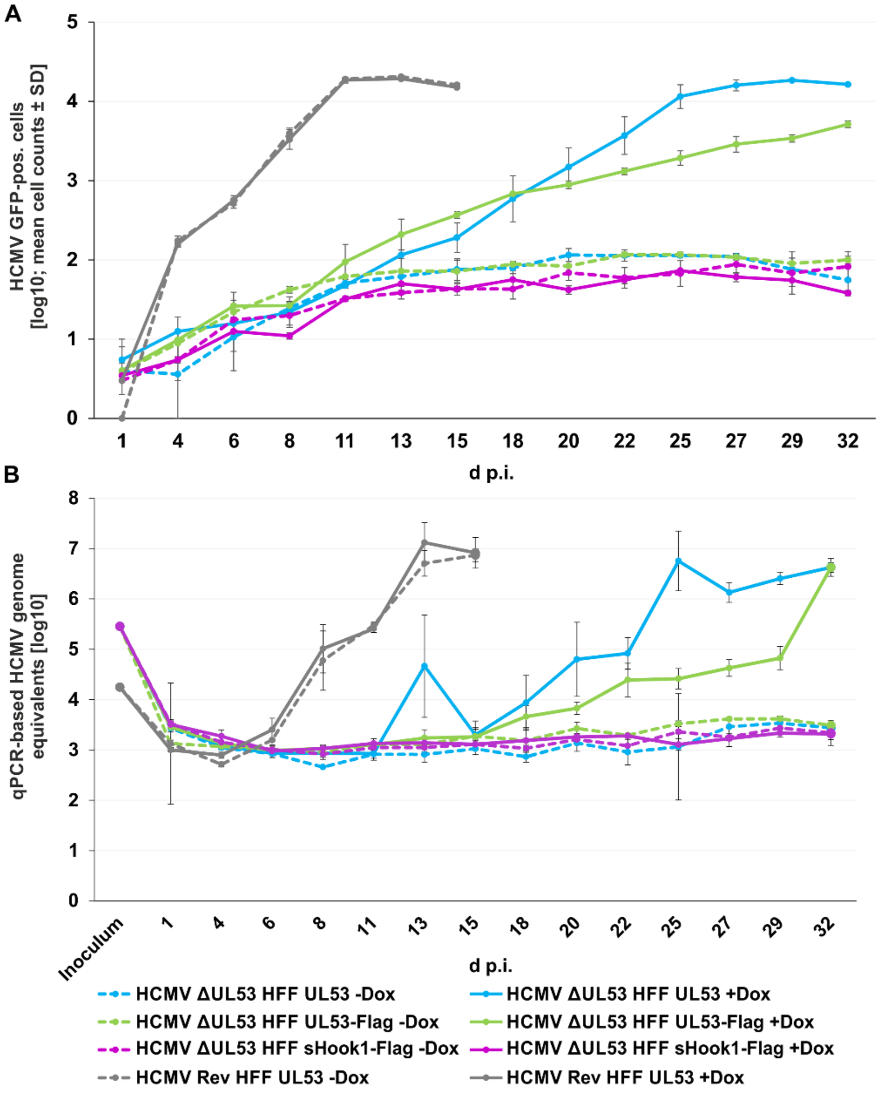

Figure S2. Viral replication kinetics of HCMV ΔUL53 and its revertant (HCMV Rev) determined by quantitation of GFP-positive cells and HCMV-specific qPCR on the different recombinant HFF populations. 80,000 inducibly expressing HFFs in 24-well plates were infected with HCMV ΔUL53 or HCMV Rev at a viral dose of 5 x 10^6 genome copies without removing the viral inoculum after adsorption. pUL53, pUL53-Flag or pUL53::sHook1-Flag protein expression was either Dox-induced (+Dox) or remained non-induced (-Dox). (**A**) The number of HCMV-infected cells was measured by detection of GFP signal-positive cells at indicated time points with the CellReporterXpress® software using the ImageXpress® Pico device. Values represent 25.04 % of the area of a well and are given as a mean value ± SD of two independently infected wells. (**B**) Viral supernatants were harvested at indicated time points and viral genome equivalents were determined by qPCR. Each value represents the mean ± SD of two independent biological replicates, each measured twice.

Table S1. Oligonucleotide primers used in this study.

Primer	Sequence (5′→ 3′)
5-BamHI-EcoRI-UL53	TAG GGATCC GAATTC ATGTCTAGCGTGAGC
3-PstI-XhoI-Stop-Flag	TAG CTGCAG CTCGAG TCA CTTGTCGTCATCGTC
5-sHook-UL53::BFLF2 Overlap aa 63-94	TTCCACAGGATCTTCCGCGCCCACTTTGAACTGGAGCTCAAGTTTCTTCG-TATGATGAAGACCCCCATCCATGGCAAAGAGTCCATCTGCTTA
3-sHook-UL53::BFLF2 Overlap aa 78-48	AAACTTGAGCTCCAGTTCAAAGTGGGCGCGGAAGATCCTGTGGAAGTCCCG-CAGCGTGAGGCGGGATCTGTCGTCGGCCGGCGACGGCGGTTC
5-sHook-UL53::Orf27 Overlap aa 63-94	CTGTTTGACATCATCGCAGAGCACCCCGAACTGGAGCTCAAGTACCTTA-GAATGATGAAGATGCCAATCATTGGCAAAGAGTCCATCTGCTTA
3-sHook-UL53::Orf27 Overlap aa 78-48	GTACTTGAGCTCCAGTTCGGGGTGCTCTGCGATGATGTCAAACAGGTCGTGG-TACACGAGCCGCTCCTTTGAGTCGGCCGGCGACGGCGGTTC
5-BFLF2 EcoRI	TAG GAATTC ATGGCCCCGGTCAC
3-BFLF2 PstI	TAG CTGCAG CTACTGTTTATTTTC
5-Orf27 EcoRI	TAG GAATTC ATGCATTTAAAG
3-Orf27 PstI	TAG CTGCAG TCACCGAGGAGGAAC
5-BFRF1 EcoRI	TAG GAATTC ATGGCGAGCCCGGAAGA
3-BFRF1(1-315) PstI	TAG CTGCAG TCAAGGTGTGGCGCGCCAAG
5-Orf24 EcoRI	TAG GAATTC ATGTCACGGAGAAC
3-Orf24(1-247) PstI	TAG CTGCAG TTAGTTAGCTACGGGTAG
5-UL53-EcoRI	TAG GAATTC ATGTCTAGCGTGAG
3-UL53(1-87)-Flag XhoI PstI	TAG CTGCAG CTCGAG CTACTTGTCGTCATCGTCTTTGTAGTC CGTGATGGCCATCTTC
5-HA-BFRF1 EcoRI	TAG GAATTC ATG TACCCATACGATGTTCCAGATTACGCT GC GAGCCCGGAAGAG
3-BFRF1(1-315) XhoI	TAG CTCGAG TCAAGGTGTGGCGCGCCAAG
5-HA-Orf24 EcoRI	TAG GAATTC ATG TACCCATACGATGTTCCAGATTACGCT TC ACGGAGAACGTATGTAC
3-Orf24(1-247) XhoI	TAG CTCGAG TTAGTTAGCTACGGGTAG
5-pUL50-1-EcoRI	TGA GAATTC ATG GAGATGAACAAGGTTCTCCATC
3-UL50(1-358) PstI	TAG CTGCAG TCACCACGGCCCAGACTG
3-UL53 PstI	TAG CTGCAG TCAAGGCGCACGAATG
5-HA-UL34 HindIII	TAGAAGCTTATGTACCCATACGATGTTCCAGATTACGCTGCGGGACTGGG-CAAGC
3-UL34 XhoI	TAGCTCGAGTTATAGGCGCGCGCCAGCAC
5-Flag-UL31 HindIII	TAGAAGCTTATGGACTACAAAGACGATGACGACAAGTATGACAC-CGACCCCCATC
3-UL31 XhoI	TAGCTCGAGCTACGGCGGAGGAAAC
5-UL53-attB1	GGGGACAAGTTTGTACAAAAAAGCAGGCTACCATGTCTAGCGTGAG
3-UL53-attB2	GGGGACCACTTTGTACAAGAAAGCTGGGTTCAAGGCGCACGAATGC
3-UL53-Flag-attB2	GGGGACCACTTTGTACAAGAAAGCTGGGTTCACTTGTCGTCATCGTCTTT-GTAGTCAGG
5′ a-b-c-Kana	GCTGGTGGACCCCACGTACGTGATAGA-CAAGTATGTCTAGTCAACAG-CATTCGTGCGCCTTAGGGATAACAGG
3′ b-c-d-Kana	TTTTCTGCCGTACAGTGTCAAGGCGCACGAATGCTGTTGACTAGACATACTT-GTCTATCAGCCAGTGTTACAACC

Table S2: Protein expression levels of HeLa and 293T cells given as mean values ± SD signal-positive cells.

		**HeLa**	**293T**
**sHook1**	**pUL50**	8±6%	26±9%
	**BFRF1**	11±3%	32±16%
	**Orf24**	24±16%	19±10%
**sHook2**	**pUL50**	10±4%	25±6%
	**BFRF1**	7±2%	44±22%
	**Orf24**	14±6%	34±7%
